# A nurse-run, pharmacist-led outpatient penicillin allergy de-label clinic in the UK

**DOI:** 10.1093/jacamr/dlag005

**Published:** 2026-02-02

**Authors:** Neil Powell, Daniel Hearsey, Tamsyn Lewis, Marie Thomas, Helen Winn, Amanda Pritchard

**Affiliations:** Pharmacy Department, Royal Cornwall Hospital, Truro TR1 3LJ, UK; School of Biomedical Sciences, University of Plymouth, Plymouth PL4 8AA, UK; Pharmacy Department, Royal Cornwall Hospital, Truro TR1 3LJ, UK; Vaccination and Health Promotion Team, Royal Cornwall Hospital, Truro TR1 3LJ, UK; Vaccination and Health Promotion Team, Royal Cornwall Hospital, Truro TR1 3LJ, UK; Vaccination and Health Promotion Team, Royal Cornwall Hospital, Truro TR1 3LJ, UK; Vaccination and Health Promotion Team, Royal Cornwall Hospital, Truro TR1 3LJ, UK

## Abstract

**Background:**

Penicillin allergy (penA) records are associated with negative patient and health-system outcomes, which makes removal of incorrect penA records (penicillin allergy de-labelling; PADL) an antimicrobial stewardship and patient safety priority. We set up a nurse-run, adult, low-risk PADL outpatient clinic, supervised by an antimicrobial pharmacist.

**Methods:**

Adult PADL guidelines were written and approved by the hospital, and PADL training was provided to nurses. Electronic adult referrals from hospital outpatient clinics and three GP surgeries in Cornwall were accepted. Patient telephone triage started from 6 January 2025, which included taking a penA-focused history, penA risk assessment and determination of PADL method. Eligible patients were invited for a direct oral penicillin challenge (DOC) test and were followed up via a telephone call 10 days after the test. The first outpatient PADL clinic was on 7 February 2025.

**Results:**

There were 404 referrals between 27 December 2024 and 16 September 2025, of which 326 were successfully contacted. Of these, 130/326 (39.9%) had a high-risk penicillin allergy history and 5/326 (1.5%) were excluded due to cognitive impairment. Of the 326 contacts, 191 (58.6%) were categorized as low risk. Of these, 22/191 (11.5%) were de-labelled on history alone, 54/191 (28.3%) were awaiting their outpatient DOC appointment or declined attending clinic, and 115/191 (60.2%) attended clinic for DOC. Of 115 patients, 110 (95.7%) patients were successfully de-labelled and 5 (4.3%) retained their allergy status due to side effects.

**Conclusions:**

PADL delivered by non-allergy nurses in the outpatient setting is safe and effective at removing low-risk penicillin allergy records.

## Introduction

Penicillin allergy (penA) records are common, but 95% of patients with low-risk penA records are able to take penicillin antibiotics after formal allergy testing.^1^ PenA records are associated with broad-spectrum antibiotic prescribing and negative patient and health-system outcomes, which makes removal of incorrect penA records (penicillin allergy de-labelling; PADL) an antimicrobial stewardship and patient safety priority.^2^ The paucity of allergy specialists worldwide has led to non-allergy healthcare worker–delivered PADL. Nurses in Hong Kong have shown they can safely deliver a direct oral challenge (DOC) of penicillin for low-risk patients in the outpatient setting, supervised by allergists.^3^ We set up a nurse-run, adult, low-risk PADL outpatient clinic, supervised by an antibiotic pharmacist.

## Methods

PADL guidelines for adult patients were written and approved by the hospital’s Medicines Practice Committee (see supplementary material). PADL training to nurses was provided by the supervising antibiotic pharmacist and included a 25 minute presentation followed by three case studies (see supplementary material). On 27 December 2024 the clinic started accepting electronic referrals for adults with penA records from the hospital outpatient clinics, inpatients not able to be tested during their inpatient stay, and three GP surgeries in Cornwall. Telephone triage of patients started on 6 January 2025. The PADL nurses attempted to contact patients via telephone on three separate occasions. Patient contact was not attempted after three failed contact attempts. Nurse telephone triage included taking a penicillin allergy-focused history, risk assessment of the penA history and determination of PADL method, if appropriate. The decision tool developed by Devchand *et al.*^4^ was used to risk assess patients. PenA risk assessments were emailed to the supervising pharmacist for a safety check before the patient was invited for a DOC test. Patients were counselled on the risk and benefits of PADL, and either emailed or posted a patient information leaflet; the latter was developed for inpatient PADL at the hospital and adapted, with permission, from guidance published by Sneddon *et al.*^5^ Patients eligible for a DOC were administered a single adult dose of the index penicillin or amoxicillin 500 mg single dose if the index penicillin was not known, after giving signed informed consent. Patients’ observations were performed pre-DOC dose and then repeated 20, 40 and 60 minutes post dose using the inpatient testing protocol, also adapted, with permission, from Sneddon *et al.*^5^ A member of the nursing team telephoned the patient 10 days after the DOC to enquire about any potential side effects from the DOC. If no symptoms were experienced, then the patient was counselled on their negative test result and to not consider themselves allergic to penicillin. If the patient experienced symptoms post testing, then those symptoms were discussed with the supervising pharmacist. If the symptoms were potentially allergic symptoms then the patient was counselled as such and the patient retained their penA status. If the symptoms were not in keeping with drug allergy, then the patient was counselled as such and a shared decision made with the patient on whether to remove their penA record. Their GP was then emailed a clinic letter to inform them of the penicillin allergy test result. The first outpatient clinic was on 7 February 2025.

## Results

Between 27 December 2024 and 16 September 2025, we received 400 referrals, of which 327 were successfully contacted and a penA-focused history taken over the telephone (see Table 1). Of these, 130/327 (39.8%) had a high-risk penicillin allergy history, and 7/327 (2.1%) were cognitively impaired and therefore excluded. One hundred and ninety of 327 (58.1%) were categorized as low risk. Of these, 21/190 (11.1%) were de-labelled on history alone, 54/190 (28.4%) either met exclusion criteria for testing, were awaiting their outpatient clinic appointment or declined attending clinic, and 115/190 (60.5%) agreed to attend clinic for DOC.

**Table 1.** Demographics of patients referred for testing

Characteristic	
Age, median (IQR), y	60 (44.5–71)
Gender, *n*	
Male	127
Female	273
Referring specialty, *n*	
Acute medicine	4
AMS pharmacist	13
Anaesthetics	16
Emergency medicine	1
Gastroenterology	3
General surgery	3
General practice	276
Gynaecology	18
Haematology	30
Infectious diseases	1
Intensive care	3
Unknown	17
Oncology	7
Orthopaedic	5
Respiratory	3
Total	400
Penicillin allergy risk category, *n*	
High risk^a^	130
Low-risk DDL^a^	21
Low-risk DOC^a^	169
Unable to obtain reliable history	7
Unable to contact patient	73
Total	400

AMS, antimicrobial stewardship; DDL, direct de-label; DOC, direct oral challenge.

^a^Risk categories as per Devchand *et al.*^4^

Of 115 patients who attended clinic for testing, 110 (95.7%) were successfully de-labelled and 5 (4.3%) retained their allergy status for the following reasons; 1 immediate rash and tachycardia (within 1 h), 2 delayed skin reactions (Day 5 and Day 3 post DOC), 1 light-headedness and nausea, and 1 gastrointestinal symptoms.

## Discussion

### Main findings

This pharmacist-led nurse-delivered outpatient PADL clinic was able to safely de-label one-third of patients who were contacted and risk assessed via telephone. Of those tested, 96% were successfully de-labelled. The reported rates of DOC-related harm were low, and comparable to the published studies.^6^

### Comparison with the literature

There is a paucity of nurse-led, low-risk PADL data.^7,8^ Kan *et al.*^9^ published a report of their allergist-led nurse-delivered outpatient clinic. Of 58 penA patients with low-risk penA histories, 56 (97%) were successfully de-labelled, a proportion comparable to our findings.^9^ In the same study, when compared with patients de-labelled via a traditional allergist pathway, a higher proportion of patients de-labelled via the nurse pathway had used penicillin for an infective episode in the 12 months post de-label.^9^ The authors postulate that this was due to the longer patient contact time in the nurse-led clinic, which enabled opportunity for patient counselling on the risks and benefits of PADL and the implications for patients of a negative result, which increases the likelihood of patients trusting their de-label status.^9^

### Strengths and weaknesses

This study risk assessed and de-labelled a large number of patients with an extended 10 day follow-up for DOC-associated side effects. It was a single-centre study and therefore our findings may not be generalizable to other hospitals in the UK. We did not follow patients up beyond 10 days post de-label to determine whether patients received penicillin antibiotics for subsequent infections or whether they were continuing to avoid penicillin post testing. We did not determine whether GP records were updated in response to the emailed PADL clinic letter.

We have not calculated staff costs to deliver the PADL clinic. Comparing the costs to deliver our model of care with the traditional allergist model of care would be useful to enable the NHS to determine implementation costs of our model of care.

### Implications for practice

There is a paucity of allergists in the UK to deliver PADL. We have demonstrated that a nurse-delivered, pharmacist-led, outpatient low-risk PADL clinic is safe and effective and therefore provides an opportunity for expanding PADL services in the NHS. Moreover, the model described here is likely less resource intensive than traditional allergist-delivered clinics, which is an important consideration in the resource limited NHS.

### Conclusions

PADL delivered by non-allergy nurses in the outpatient setting is safe and effective at removing low-risk penicillin allergy records.

## Supplementary Material

dlag005_Supplementary_Data

## Supplementary material

Hospital penicillin allergy de-labelling guidelines and penicillin allergy de-labelling training material are available as Supplemntary data at *JAC-AMR* Online.
