# Supplementary material for: A nurse-run, pharmacist-led outpatient penicillin allergy de-label clinic in the UK
Source: JAC Antimicrob Resist. 2026 Feb 2;8(1):dlag005. doi: 10.1093/jacamr/dlag005 (PMC12862639; doi:10.1093/jacamr/dlag005)
Supplement: dlag005_Supplementary_Data [file dlag005_supplementary_data.zip › Case study 1_patient brief_PMOS.docx]

**Case study 1 - patient brief**

Peter is a 21-year-old male

**Penicillin allergy history questions and answers**

**Which penicillin did you react to?**

*Don’t know, is there more than one kind?*

**What were the details of the reaction/what happened to you?**

*I had penicillin from my GP when I was young, maybe 4 or 5 years old. My mum said that it made me sick and I vomited every time I took it*

**How many hours after having your first dose of the antibiotic did the reaction occur?**

*I’m not sure, I think I vomited it up straight away. I think I just didn’t like the taste of it.*

**How many years ago did the reaction occur?**

*Well, I suppose it’s fifteen years ago now.*

**How was the reaction managed? Did you need to go a hospital for treatment?**

*No*

**Which other antibiotics have you tolerated post reaction (to check if the index penicillin or amoxicillin has since been tolerated)?**

*I am not sure I have had any antibiotics since then.*
