# Supplementary material for: A nurse-run, pharmacist-led outpatient penicillin allergy de-label clinic in the UK
Source: JAC Antimicrob Resist. 2026 Feb 2;8(1):dlag005. doi: 10.1093/jacamr/dlag005 (PMC12862639; doi:10.1093/jacamr/dlag005)
Supplement: dlag005_Supplementary_Data [file dlag005_supplementary_data.zip › Case study 2 HCW copy_PMOS.docx]

The aim is to elicit a penicillin allergy focused history from the patient and then, if appropriate, de-label the patient describing the steps you would undertake in the de-label process.

**Case study 2**

Mr Geraint Williams is an 80-year-old male

Which penicillin did you react to?

What were the details of the reaction/what happened to you?

How many hours after having your first dose of the antibiotic did the reaction occur?

How many years ago did the reaction occur?

How was the reaction managed? Did you need to go a hospital for treatment?

Which other antibiotics have you tolerated post reaction (to check if the index penicillin or amoxicillin has since been tolerated)? Had flucloxacillin last year from GP records

- **Which penA risk category would you put this patient in?**
- **Counsel on risks and benefits of de-label**
- **Describe the penA de-label process and steps you would take.**
