# Supplementary material for: A nurse-run, pharmacist-led outpatient penicillin allergy de-label clinic in the UK
Source: JAC Antimicrob Resist. 2026 Feb 2;8(1):dlag005. doi: 10.1093/jacamr/dlag005 (PMC12862639; doi:10.1093/jacamr/dlag005)
Supplement: dlag005_Supplementary_Data [file dlag005_supplementary_data.zip › Case study 2 patient copy_PMOS.docx]

**Case study 2**

Mr Geraint Williams is an 80-year-old male patient

**Which penicillin did you react to?**

Doesn’t know which penicillin, just that it was penicillin

**What were the details of the reaction/what happened to you?**

Unsure, occurred when a child.

**How many hours after having your first dose of the antibiotic did the reaction occur?**

Doesn’t know.

**How many years ago did the reaction occur?**

When he was a child, guesses maybe 5 years old.

**How was the reaction managed? Did you need to go a hospital for treatment?**

Doesn’t know how managed.

**Which other antibiotics have you tolerated post reaction (to check if the index penicillin or amoxicillin has since been tolerated)?**

He hasn’t received many antibiotics. He does recall having an antibiotic last year from the GP but unsure of the name.

- **Which penA risk category would you put this patient in?**
- **Counsel on risks and benefits of de-label**
- **Describe the penA de-label process to the patient (i.e. next steps).**
