# Supplementary material for: A nurse-run, pharmacist-led outpatient penicillin allergy de-label clinic in the UK
Source: JAC Antimicrob Resist. 2026 Feb 2;8(1):dlag005. doi: 10.1093/jacamr/dlag005 (PMC12862639; doi:10.1093/jacamr/dlag005)
Supplement: dlag005_Supplementary_Data [file dlag005_supplementary_data.zip › case study 3 patient brief_PMOS.docx]

**Case study 3 – patient brief**

Alec O’Neill is a 76-year-old male

**Which penicillin did you react to?**

“So I am not one hundred percent sure, but I think it may have been penicillin V.”

**What were the details of the reaction/what happened to you?**

“I know it was quite awful really, I couldn’t breathe properly and had a red blotchy rash.”

**How many hours after having your first dose of the antibiotic did the reaction occur?**

“So, after the first dose, I started getting a blotchy rash and my breathing worsened. I think I was being treated for asthma and I may have also had pneumonia at the time.”

**How many years ago did the reaction occur?**

“It was 71 years ago, if I am not mistaken.”

**How was the reaction managed? Did you need to go a hospital for treatment?**

“I stopped taking the antibiotic immediately and was also given a calamine lotion for the rash.”

**Which other antibiotics have you tolerated post reaction (to check if the index penicillin or amoxicillin has since been tolerated)?**

“So, I last tried penicillin again about 20 years ago when my GP trialled me on the antibiotic. Again, my throat tightened and had the same blotchy rash as when I was younger. I definitely do not want to try penicillin again!”
