# Supplementary material for: A nurse-run, pharmacist-led outpatient penicillin allergy de-label clinic in the UK
Source: JAC Antimicrob Resist. 2026 Feb 2;8(1):dlag005. doi: 10.1093/jacamr/dlag005 (PMC12862639; doi:10.1093/jacamr/dlag005)
Supplement: dlag005_Supplementary_Data [file dlag005_supplementary_data.zip › Compencies part 1_v7.2_080524.pptx]

## Slide 1
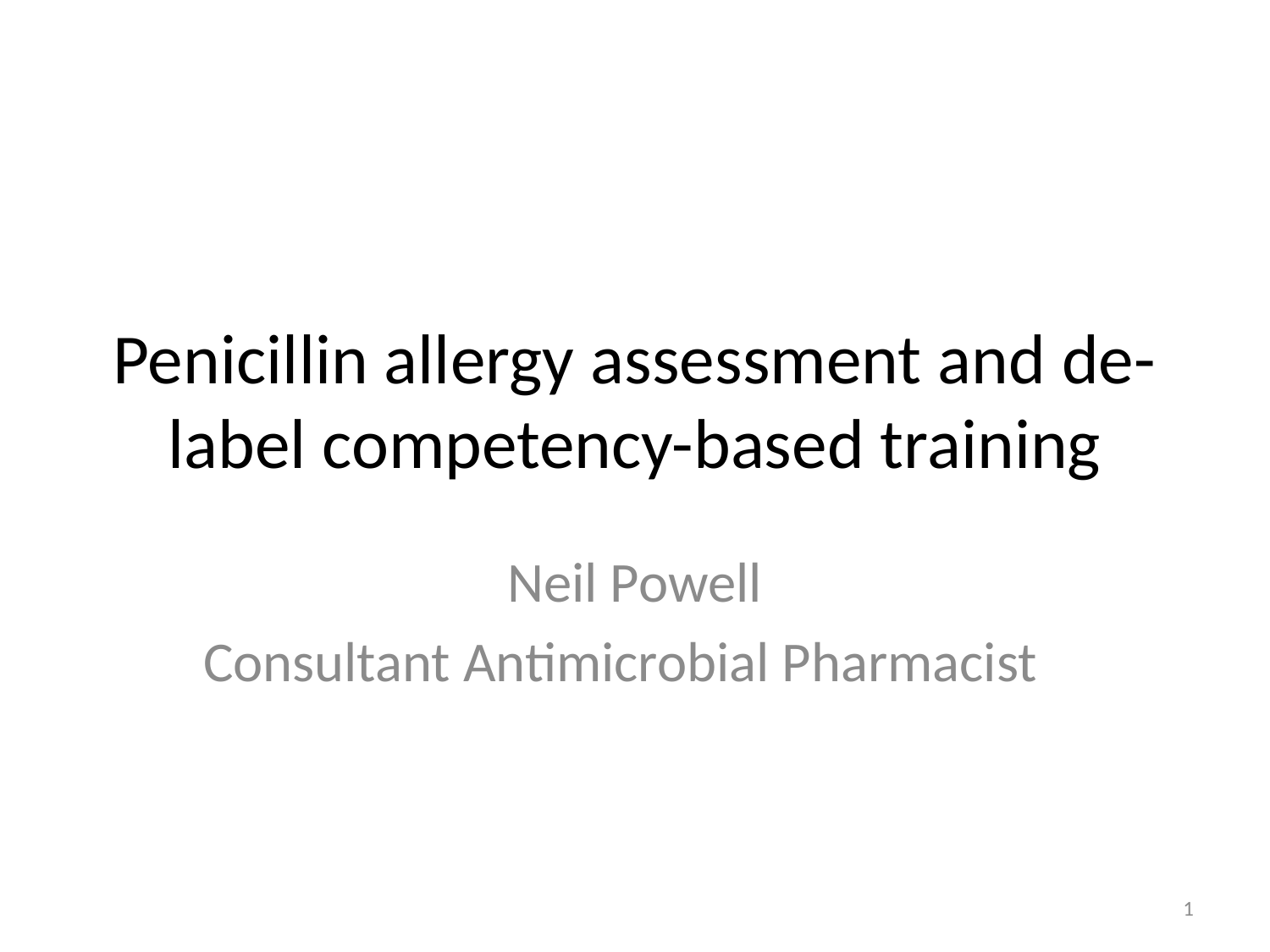

# Penicillin allergy assessment and de-label competency-based training
Neil Powell
Consultant Antimicrobial Pharmacist
1

## Slide 2
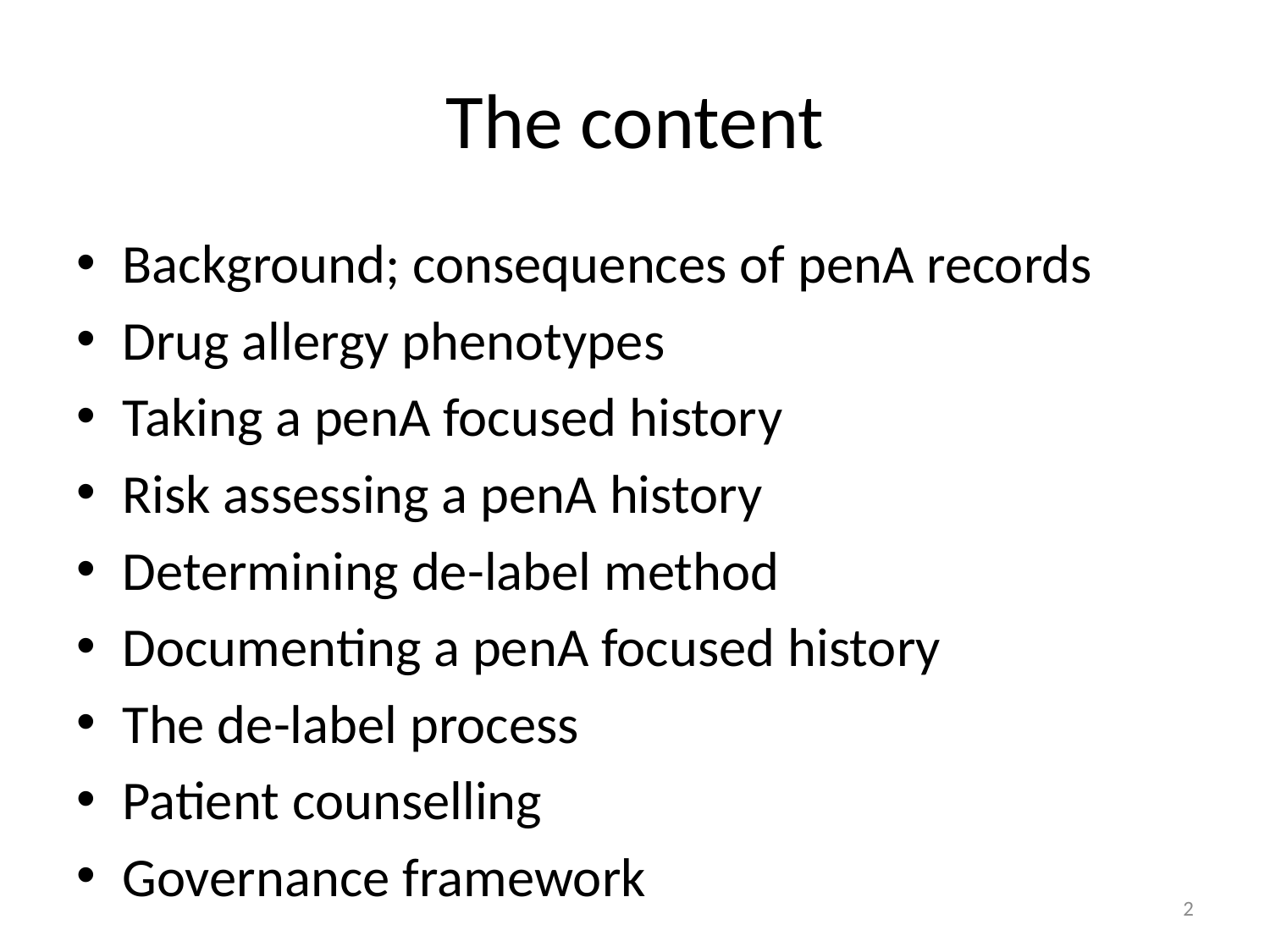

# The content
Background; consequences of penA records
Drug allergy phenotypes
Taking a penA focused history
Risk assessing a penA history
Determining de-label method
Documenting a penA focused history
The de-label process
Patient counselling
Governance framework
2

## Slide 3
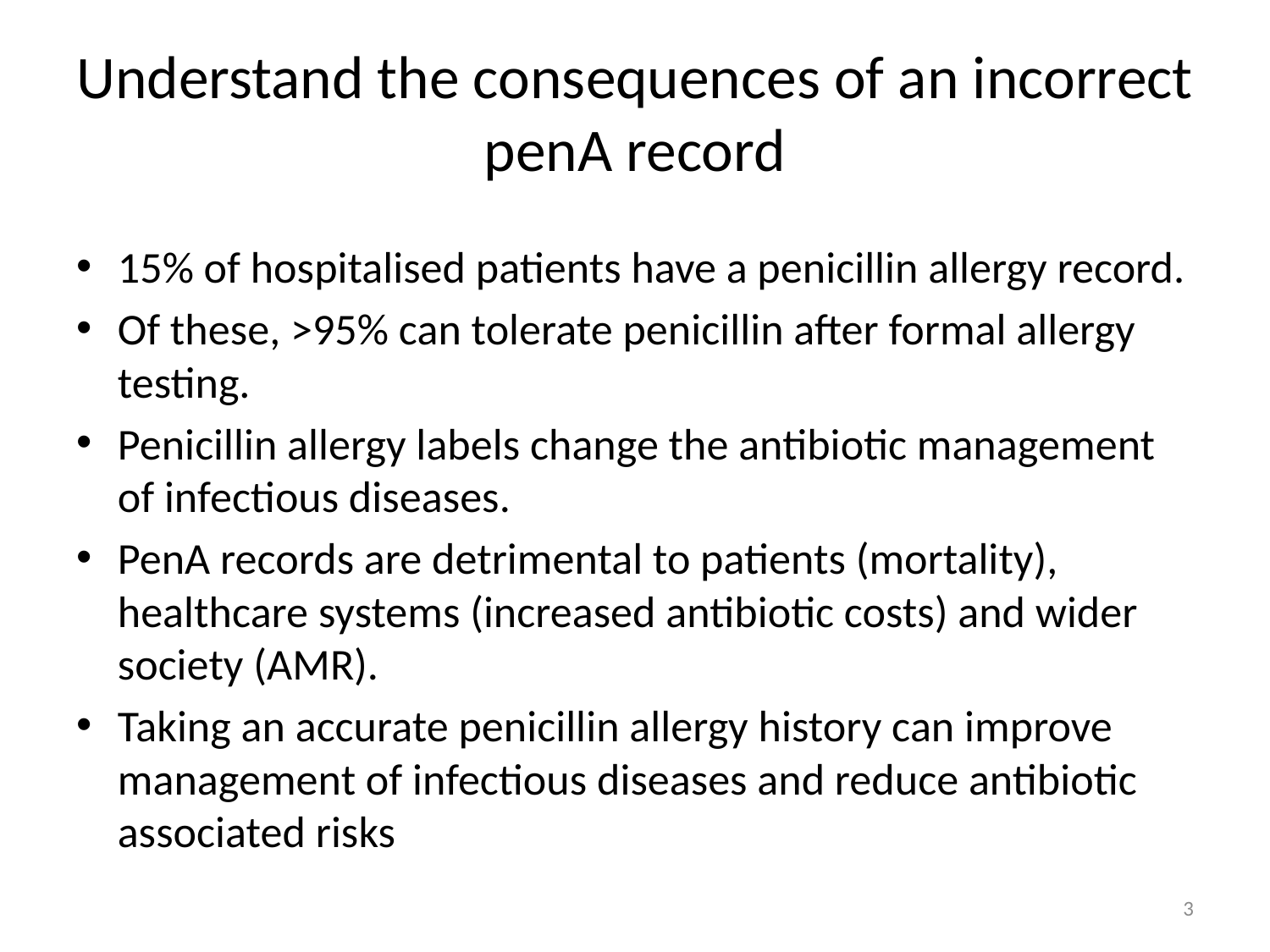

# Understand the consequences of an incorrect penA record
15% of hospitalised patients have a penicillin allergy record.
Of these, >95% can tolerate penicillin after formal allergy testing.
Penicillin allergy labels change the antibiotic management of infectious diseases.
PenA records are detrimental to patients (mortality), healthcare systems (increased antibiotic costs) and wider society (AMR).
Taking an accurate penicillin allergy history can improve management of infectious diseases and reduce antibiotic associated risks
3

## Slide 4
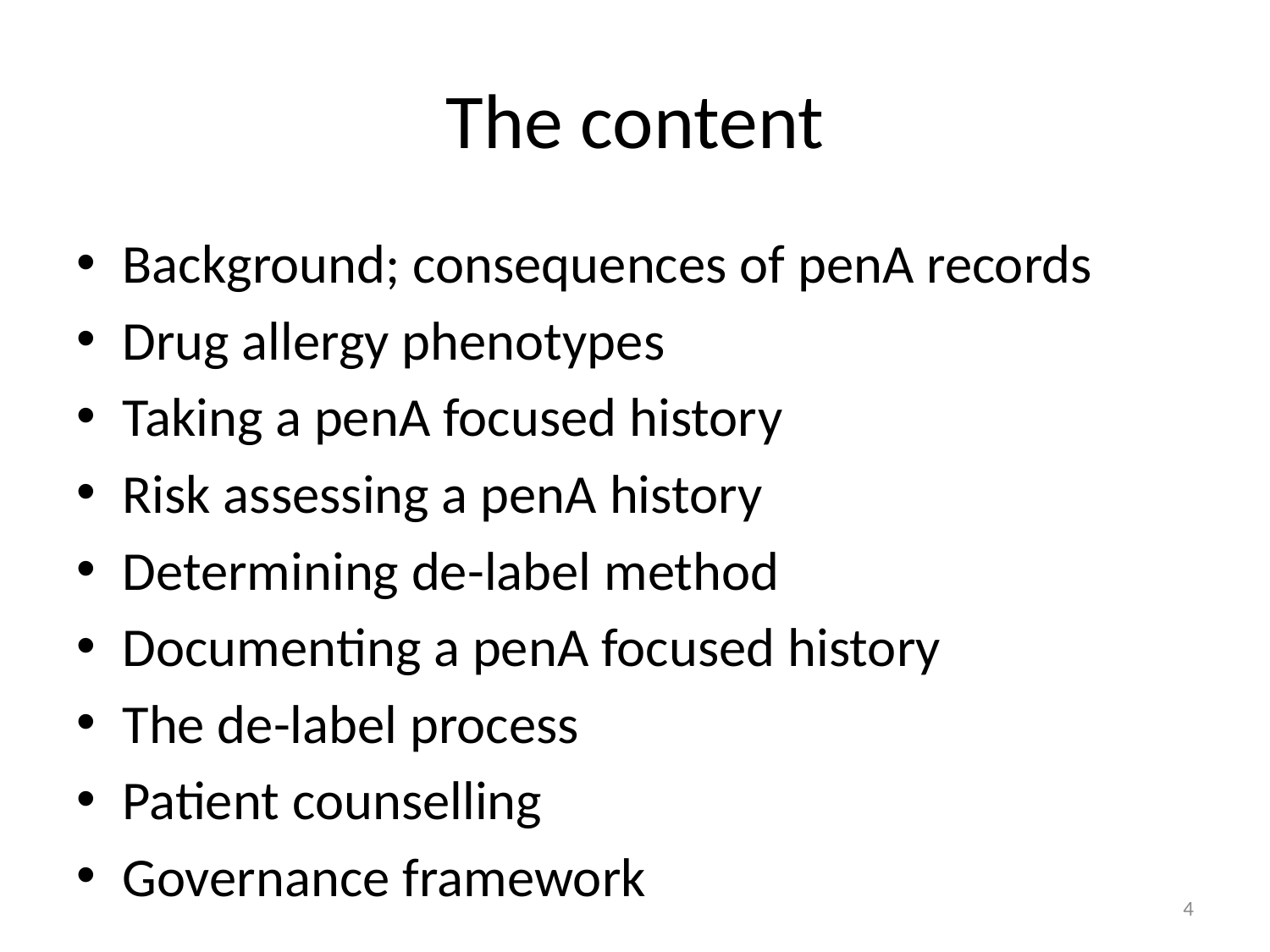

# The content
Background; consequences of penA records
Drug allergy phenotypes
Taking a penA focused history
Risk assessing a penA history
Determining de-label method
Documenting a penA focused history
The de-label process
Patient counselling
Governance framework
4

## Slide 5
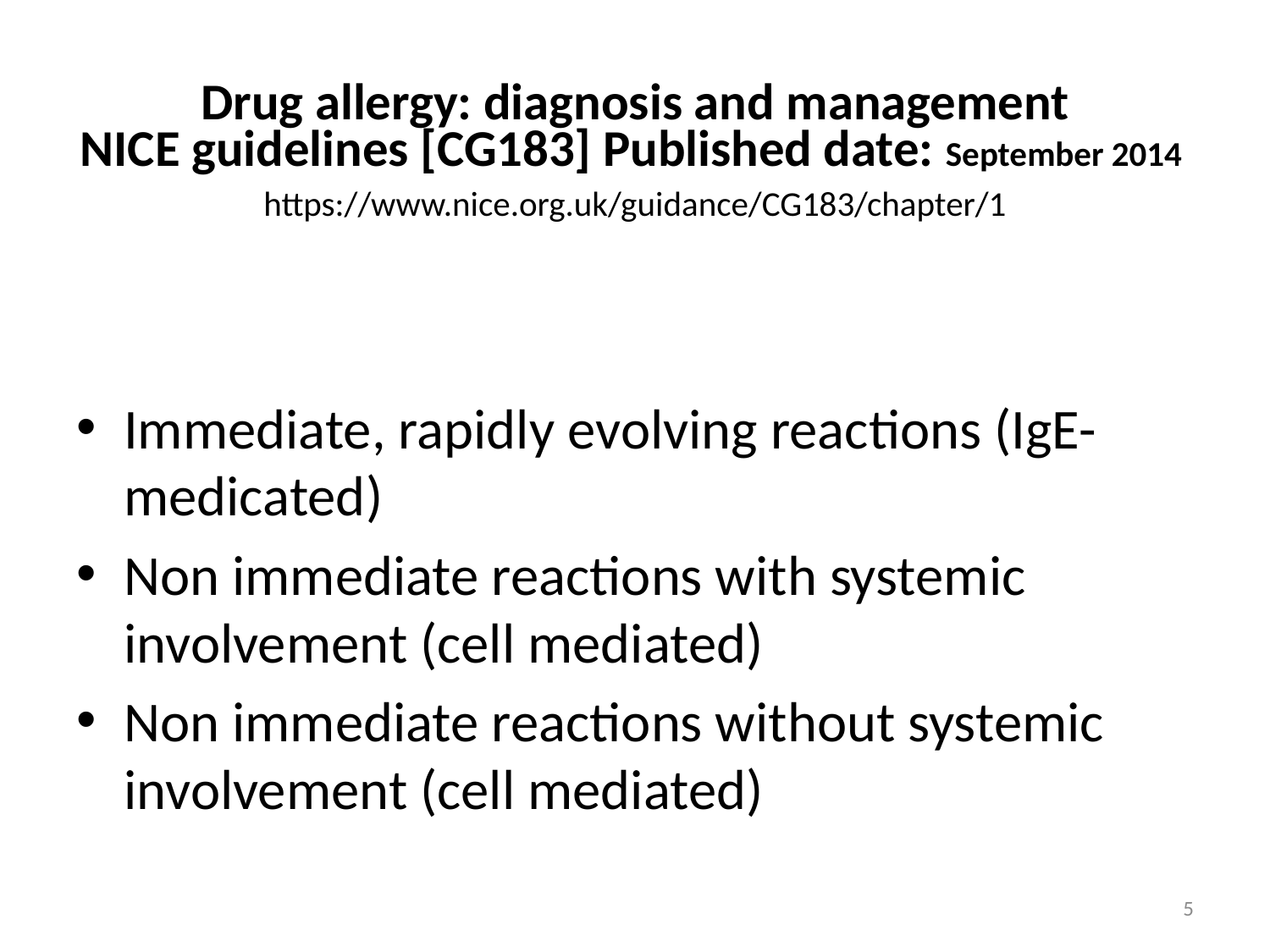

# Drug allergy: diagnosis and management NICE guidelines [CG183] Published date: September 2014 https://www.nice.org.uk/guidance/CG183/chapter/1
Immediate, rapidly evolving reactions (IgE-medicated)
Non immediate reactions with systemic involvement (cell mediated)
Non immediate reactions without systemic involvement (cell mediated)
5

## Slide 6
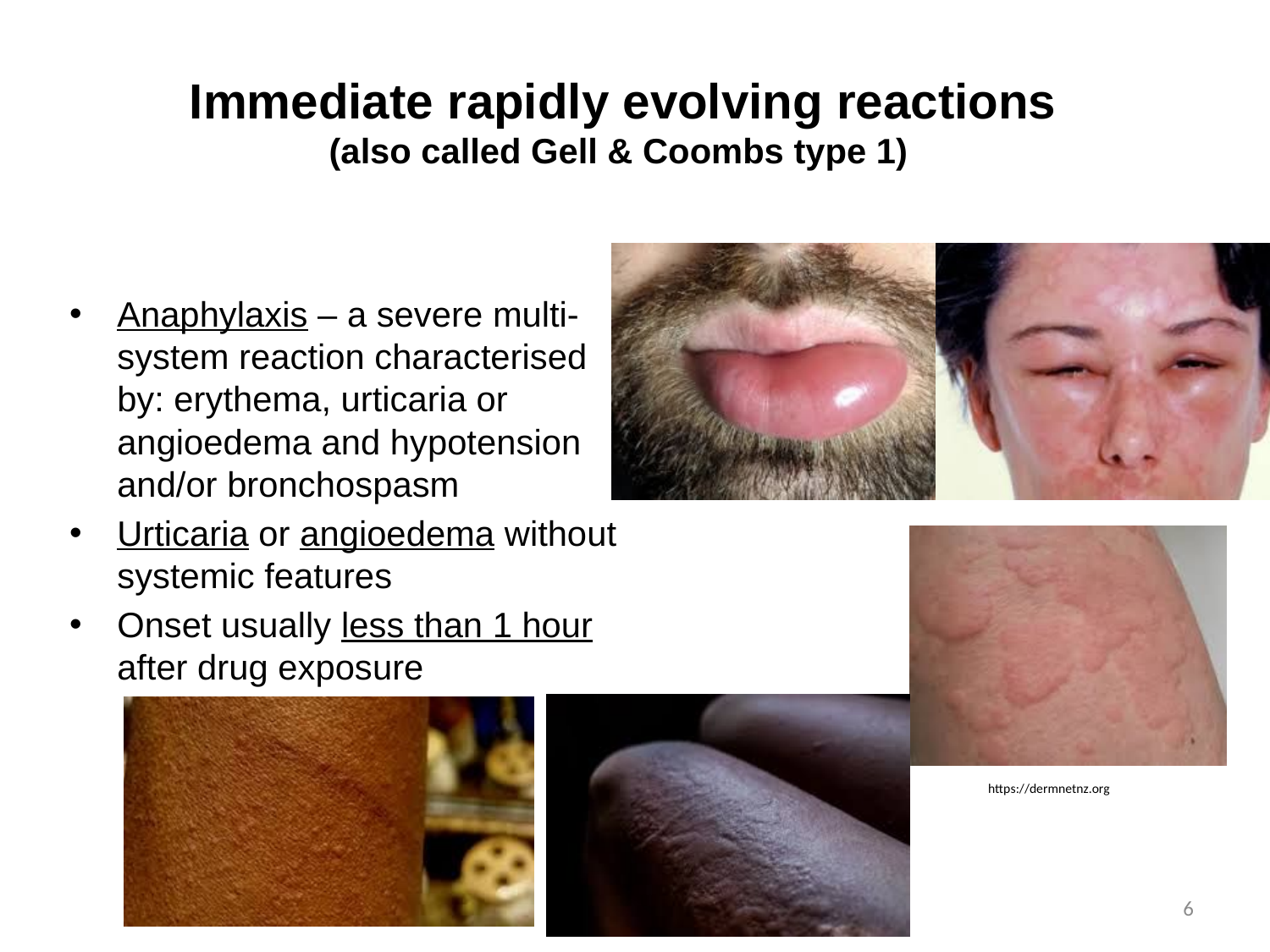

# Immediate rapidly evolving reactions (also called Gell & Coombs type 1)
Anaphylaxis – a severe multi-system reaction characterised by: erythema, urticaria or angioedema and hypotension and/or bronchospasm
Urticaria or angioedema without systemic features
Onset usually less than 1 hour after drug exposure
https://dermnetnz.org
6

## Slide 7
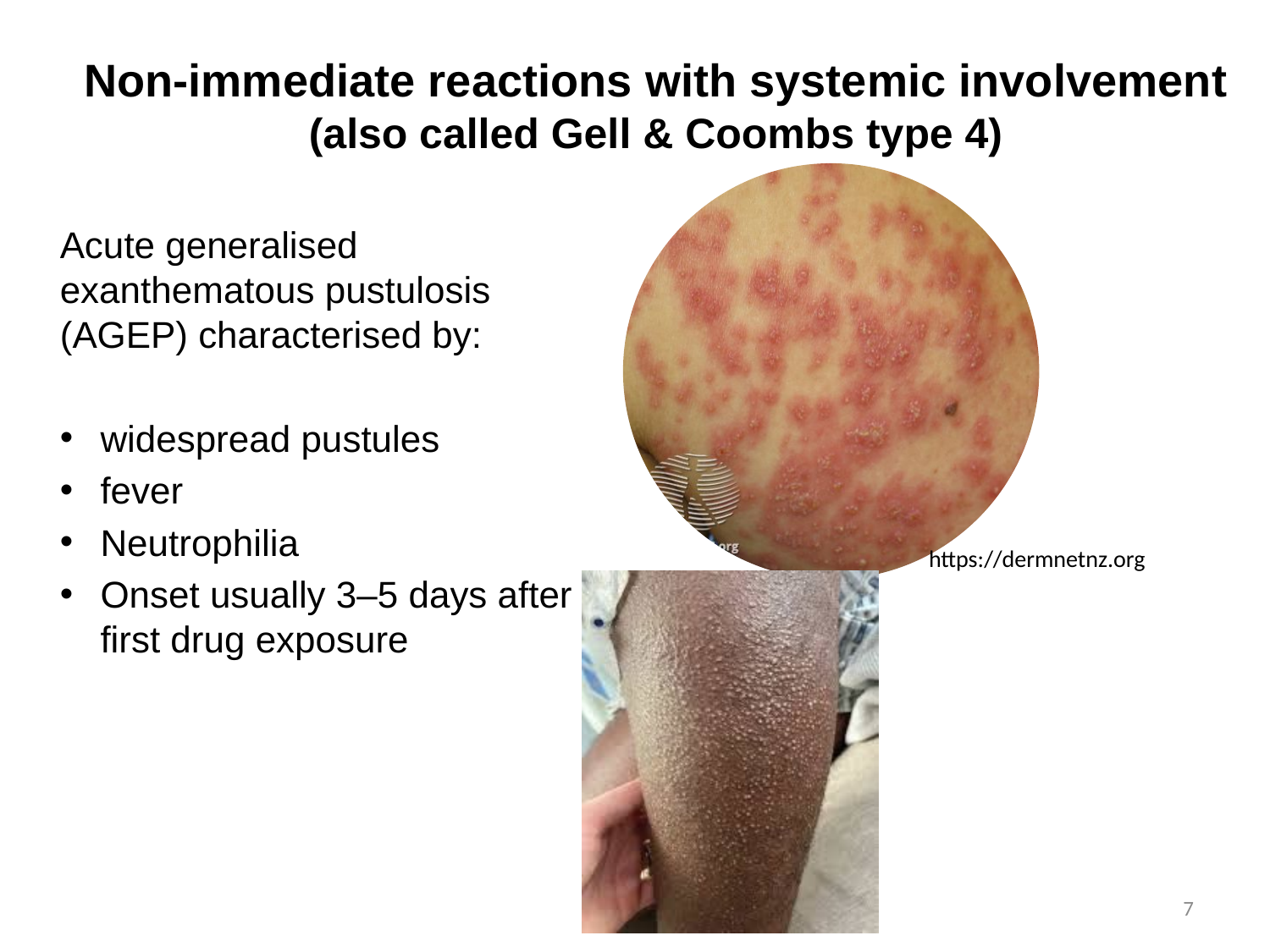

# Non-immediate reactions with systemic involvement (also called Gell & Coombs type 4)
Acute generalised exanthematous pustulosis (AGEP) characterised by:
widespread pustules
fever
Neutrophilia
Onset usually 3–5 days after first drug exposure
https://dermnetnz.org
7

## Slide 8
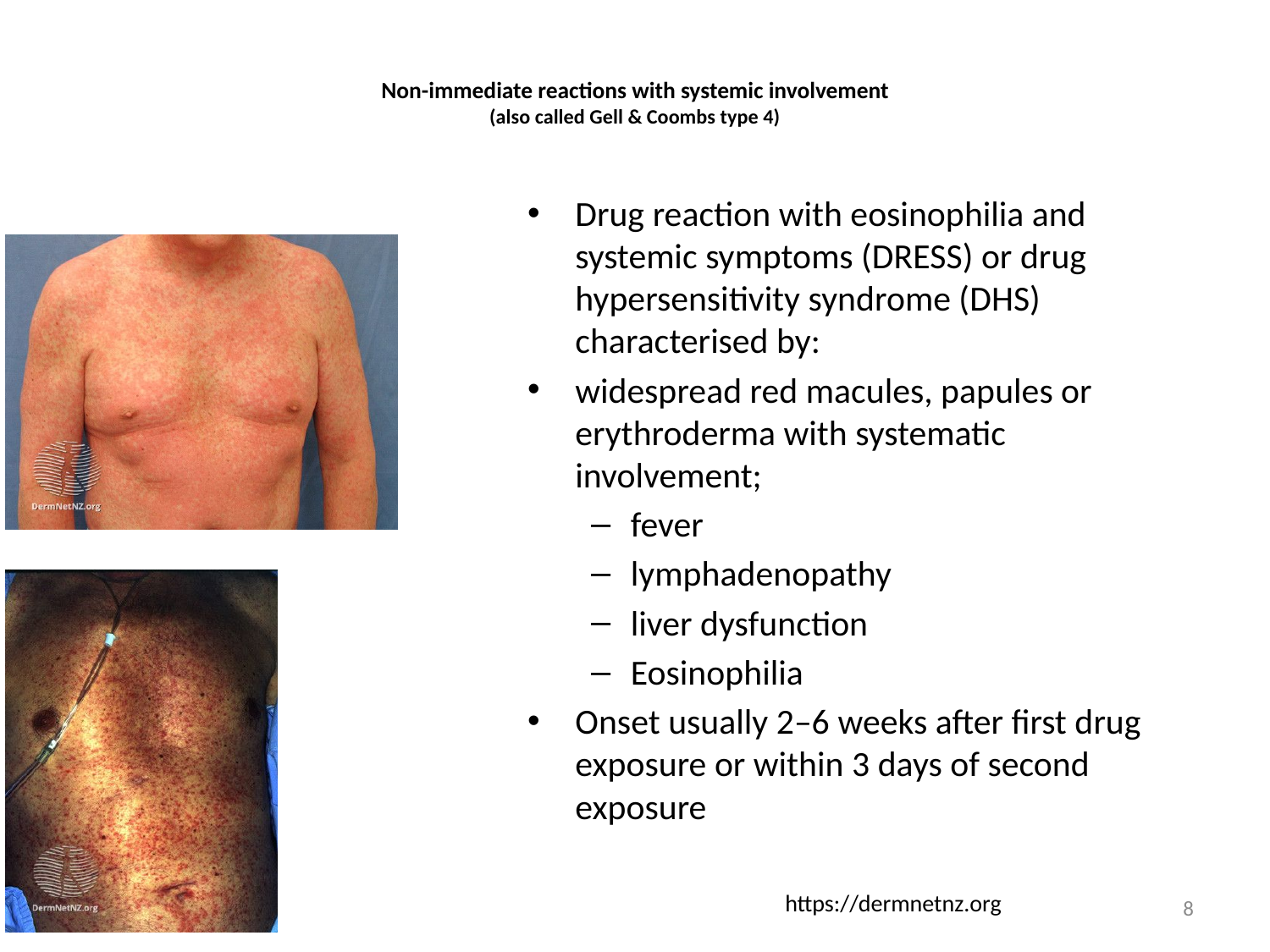

# Non-immediate reactions with systemic involvement (also called Gell & Coombs type 4)
Drug reaction with eosinophilia and systemic symptoms (DRESS) or drug hypersensitivity syndrome (DHS) characterised by:
widespread red macules, papules or erythroderma with systematic involvement;
fever
lymphadenopathy
liver dysfunction
Eosinophilia
Onset usually 2–6 weeks after first drug exposure or within 3 days of second exposure
https://dermnetnz.org
8

## Slide 9
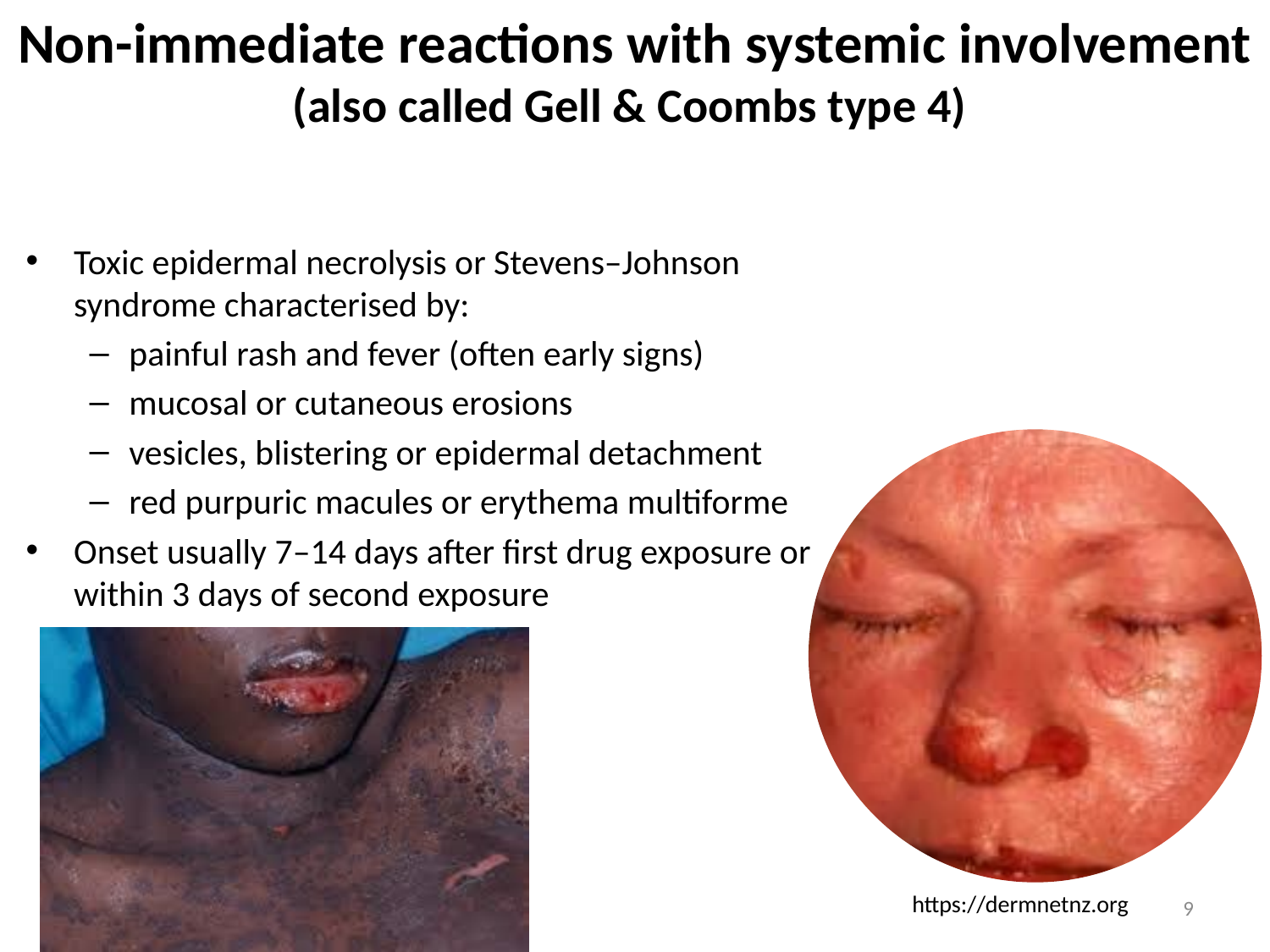

# Non-immediate reactions with systemic involvement (also called Gell & Coombs type 4)
Toxic epidermal necrolysis or Stevens–Johnson syndrome characterised by:
painful rash and fever (often early signs)
mucosal or cutaneous erosions
vesicles, blistering or epidermal detachment
red purpuric macules or erythema multiforme
Onset usually 7–14 days after first drug exposure or within 3 days of second exposure
https://dermnetnz.org
9

## Slide 10
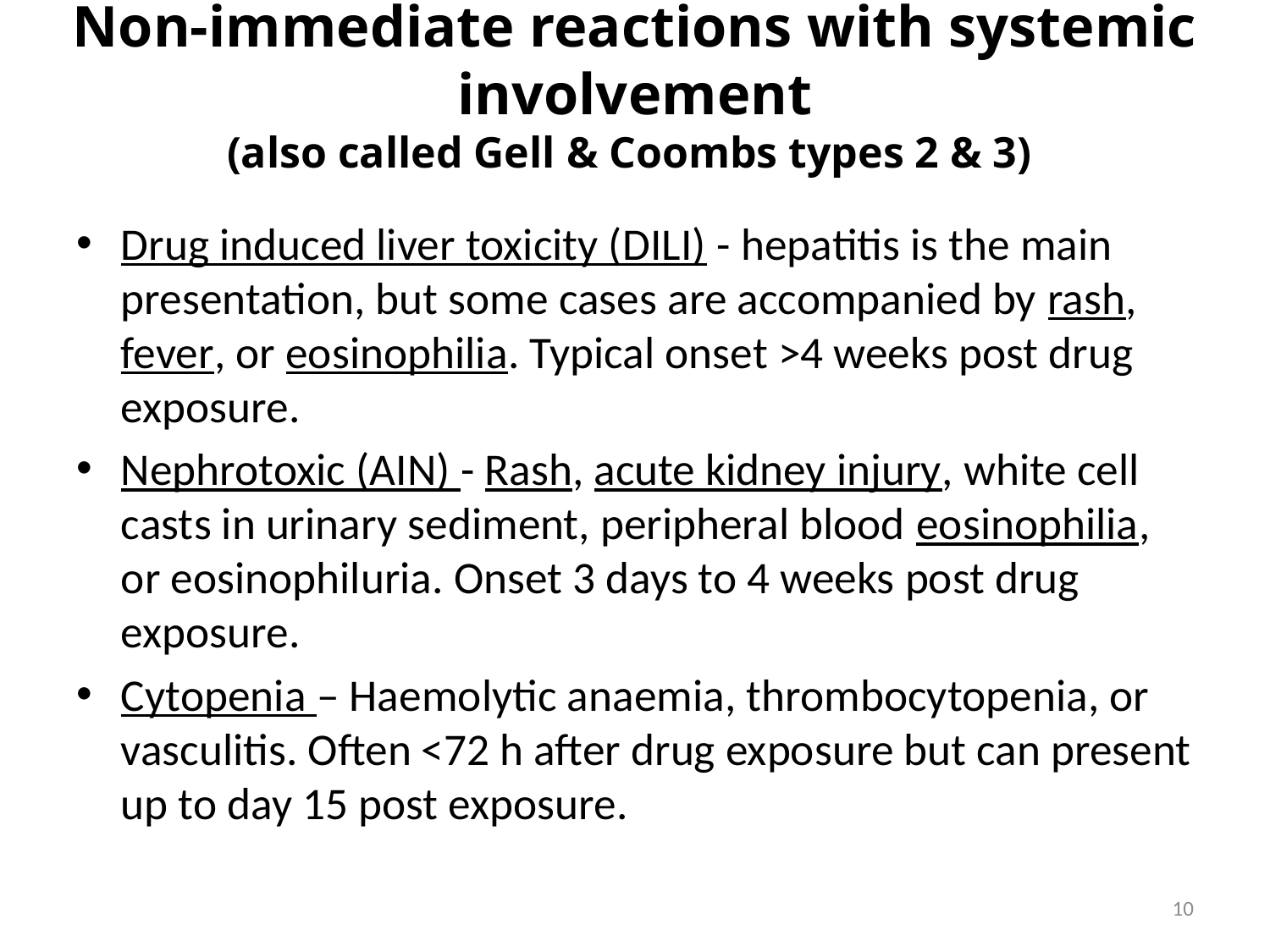

# Non-immediate reactions with systemic involvement (also called Gell & Coombs types 2 & 3)
Drug induced liver toxicity (DILI) - hepatitis is the main presentation, but some cases are accompanied by rash, fever, or eosinophilia. Typical onset >4 weeks post drug exposure.
Nephrotoxic (AIN) - Rash, acute kidney injury, white cell casts in urinary sediment, peripheral blood eosinophilia, or eosinophiluria. Onset 3 days to 4 weeks post drug exposure.
Cytopenia – Haemolytic anaemia, thrombocytopenia, or vasculitis. Often <72 h after drug exposure but can present up to day 15 post exposure.
10

## Slide 11
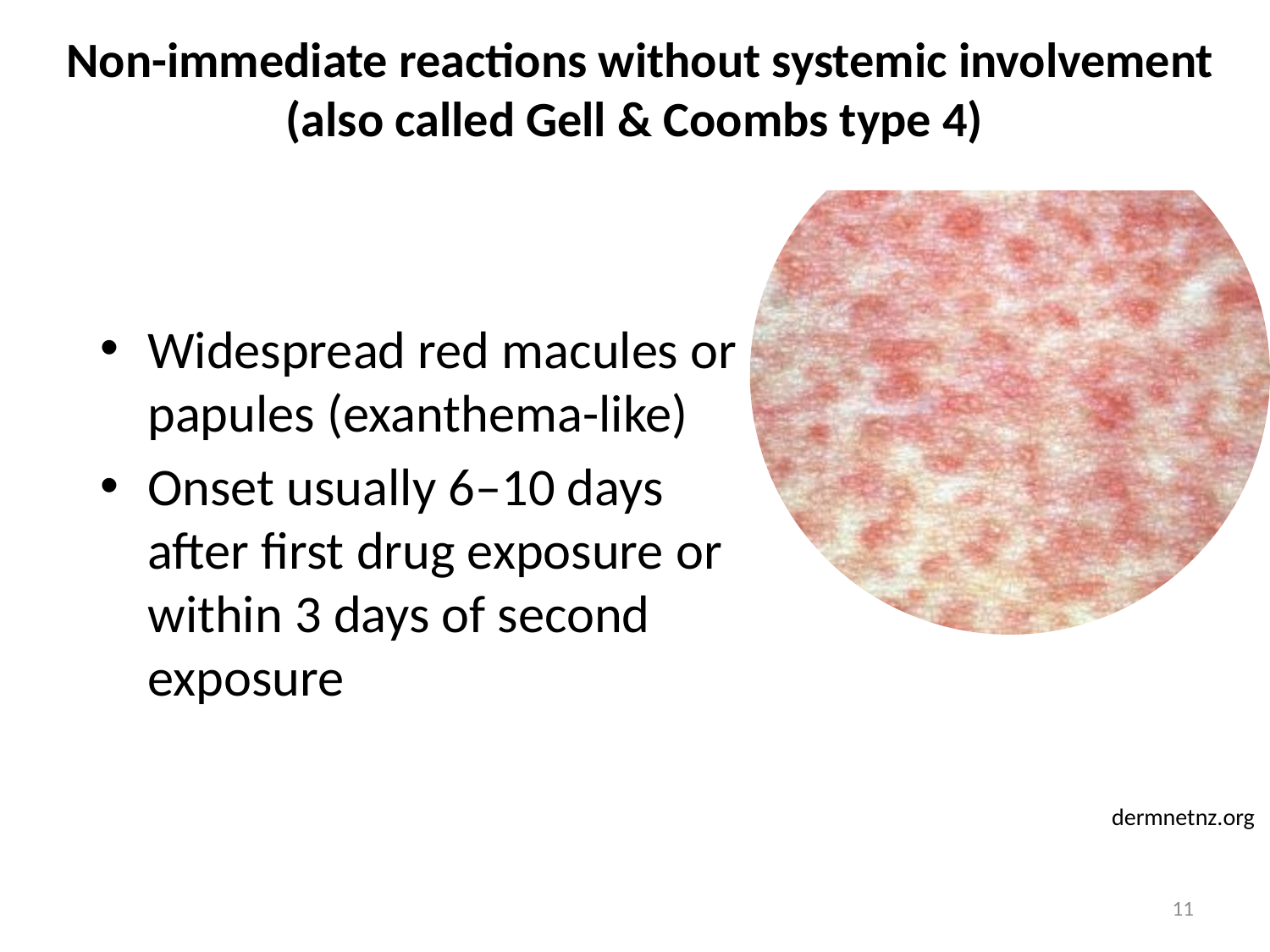

# Non-immediate reactions without systemic involvement (also called Gell & Coombs type 4)
Widespread red macules or papules (exanthema-like)
Onset usually 6–10 days after first drug exposure or within 3 days of second exposure
dermnetnz.org
11

## Slide 12
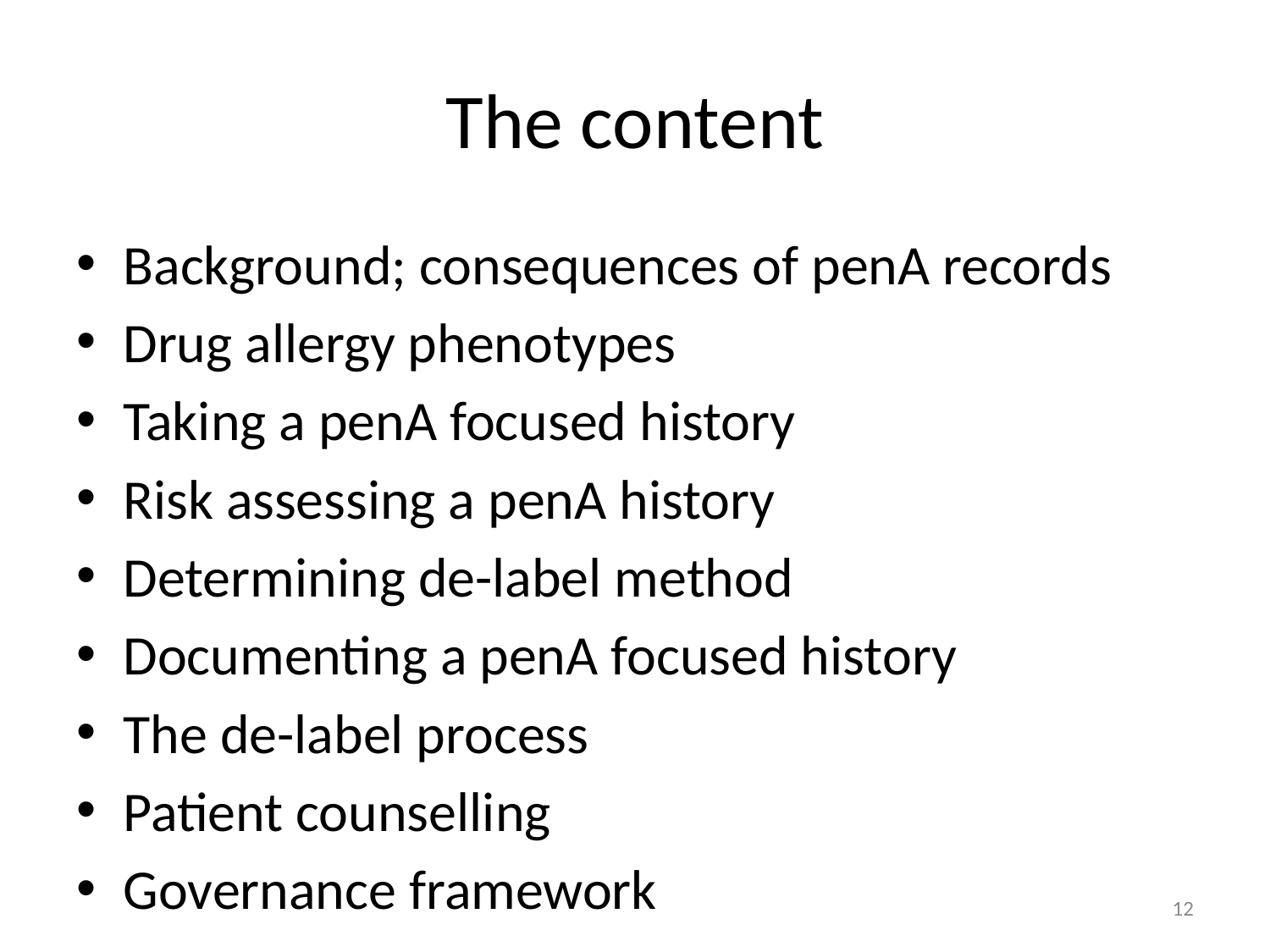

# The content
Background; consequences of penA records
Drug allergy phenotypes
Taking a penA focused history
Risk assessing a penA history
Determining de-label method
Documenting a penA focused history
The de-label process
Patient counselling
Governance framework
12

## Slide 13
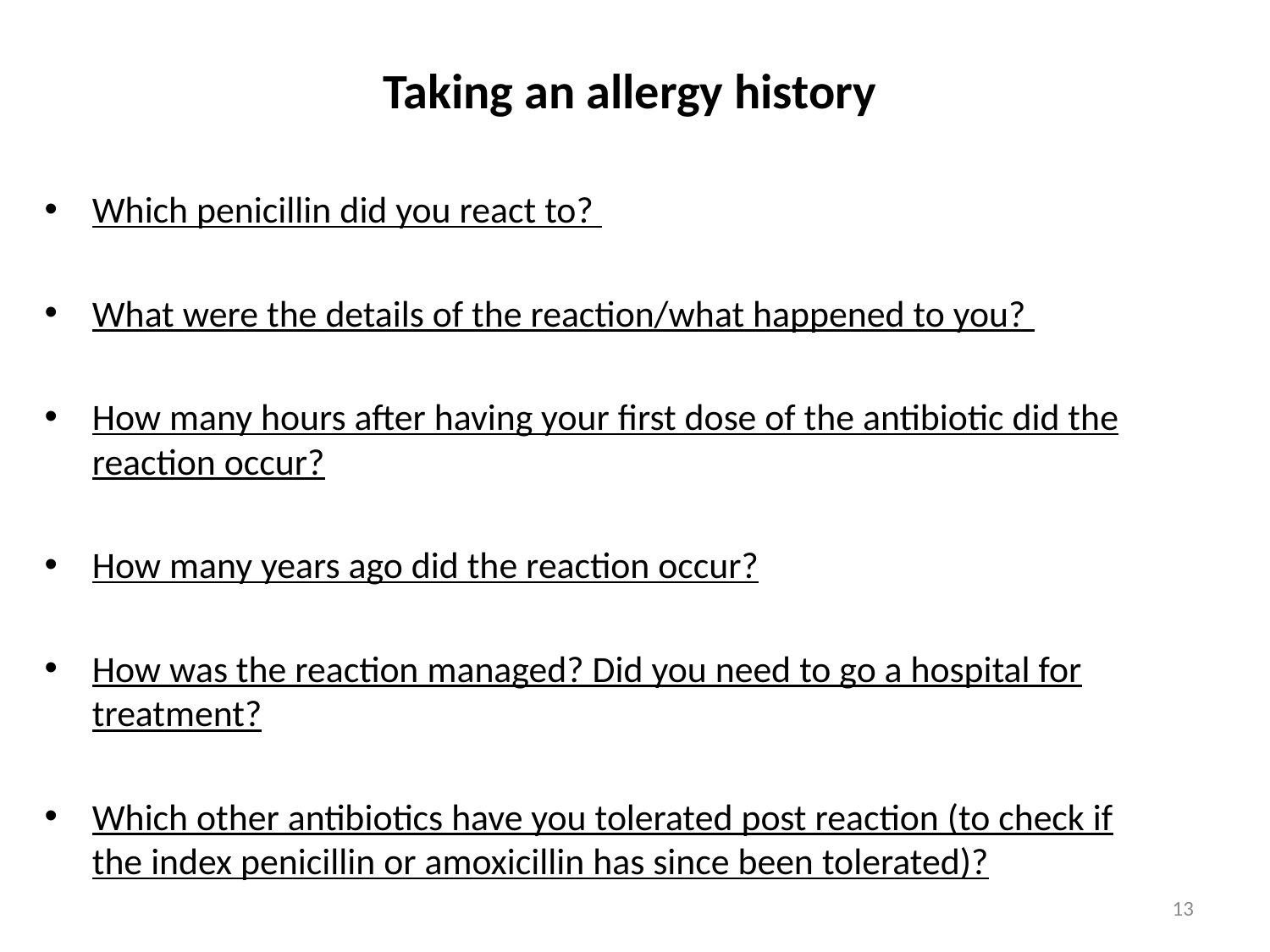

Taking an allergy history
Which penicillin did you react to?
What were the details of the reaction/what happened to you?
How many hours after having your first dose of the antibiotic did the reaction occur?
How many years ago did the reaction occur?
How was the reaction managed? Did you need to go a hospital for treatment?
Which other antibiotics have you tolerated post reaction (to check if the index penicillin or amoxicillin has since been tolerated)?
13

## Slide 14
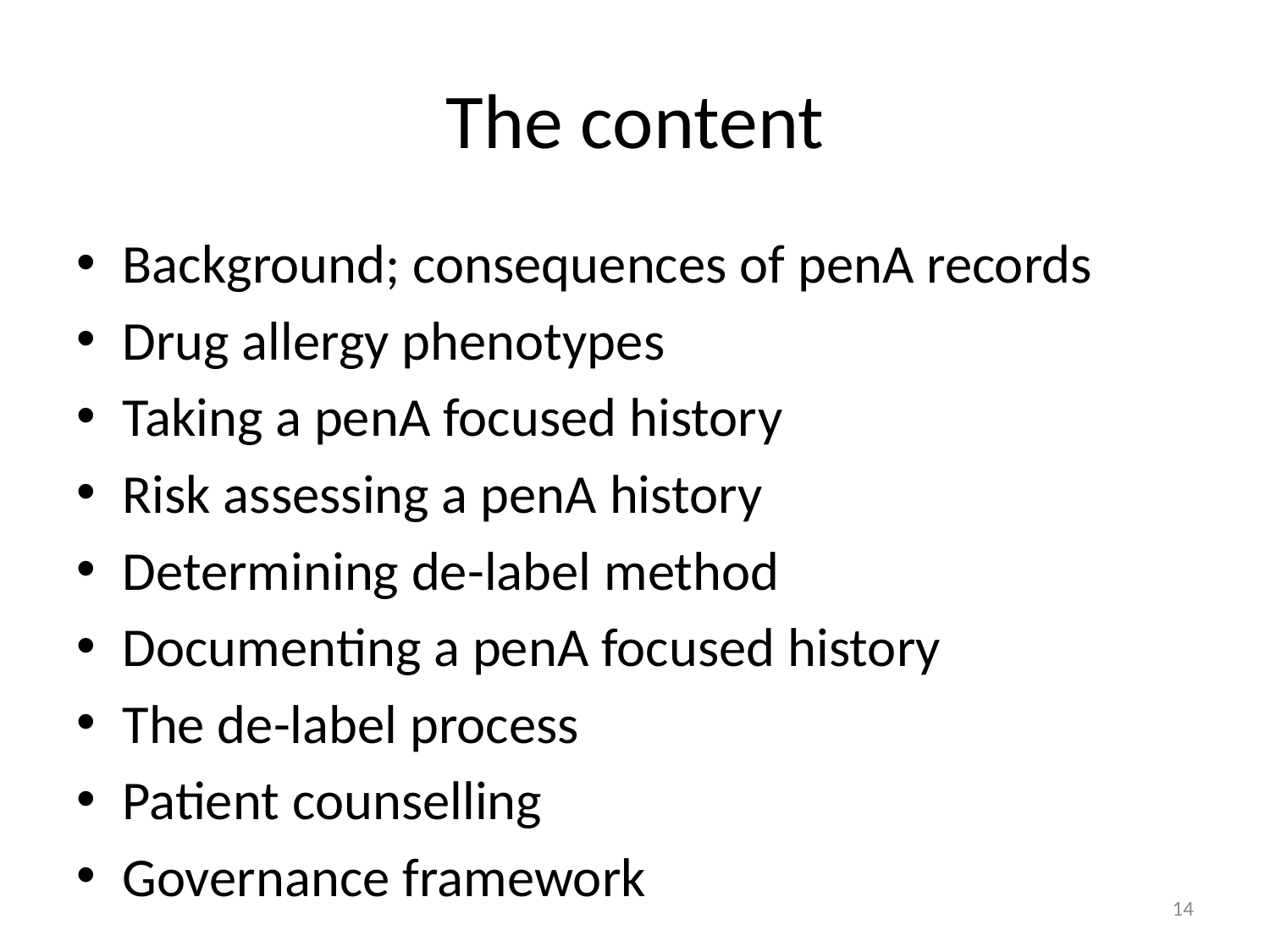

# The content
Background; consequences of penA records
Drug allergy phenotypes
Taking a penA focused history
Risk assessing a penA history
Determining de-label method
Documenting a penA focused history
The de-label process
Patient counselling
Governance framework
14

## Slide 15
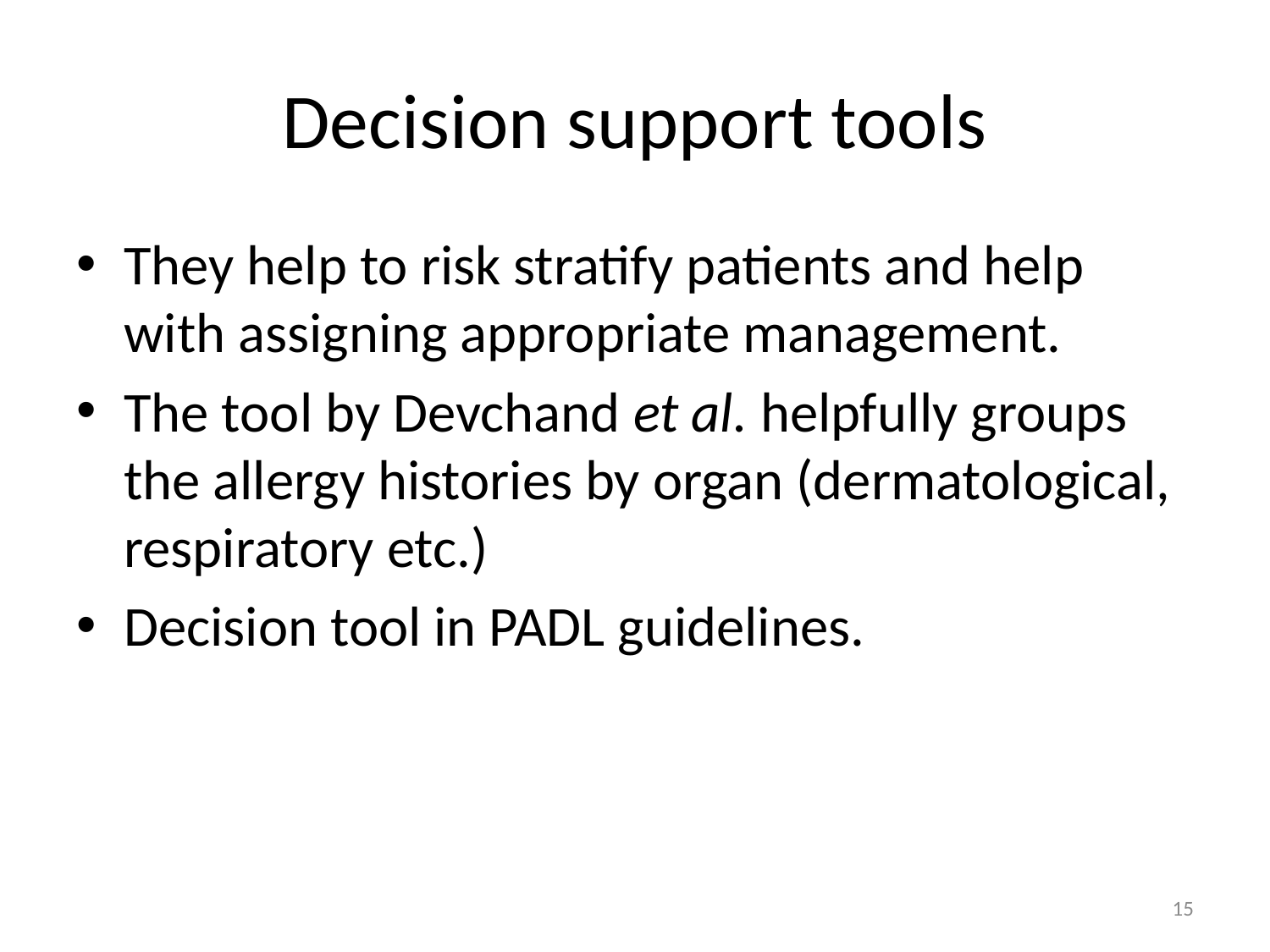

# Decision support tools
They help to risk stratify patients and help with assigning appropriate management.
The tool by Devchand et al. helpfully groups the allergy histories by organ (dermatological, respiratory etc.)
Decision tool in PADL guidelines.
15

## Slide 16
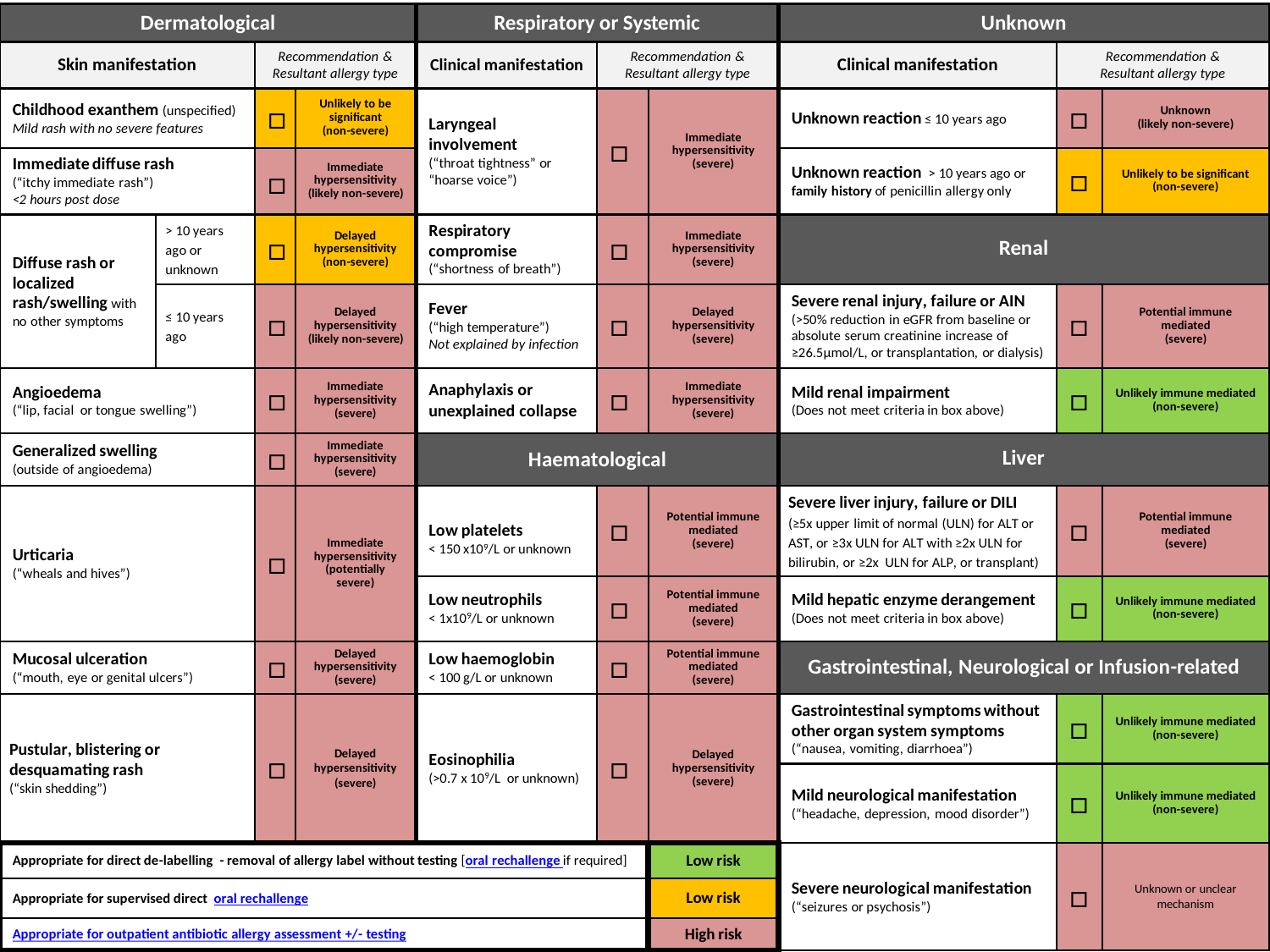

16

## Slide 17
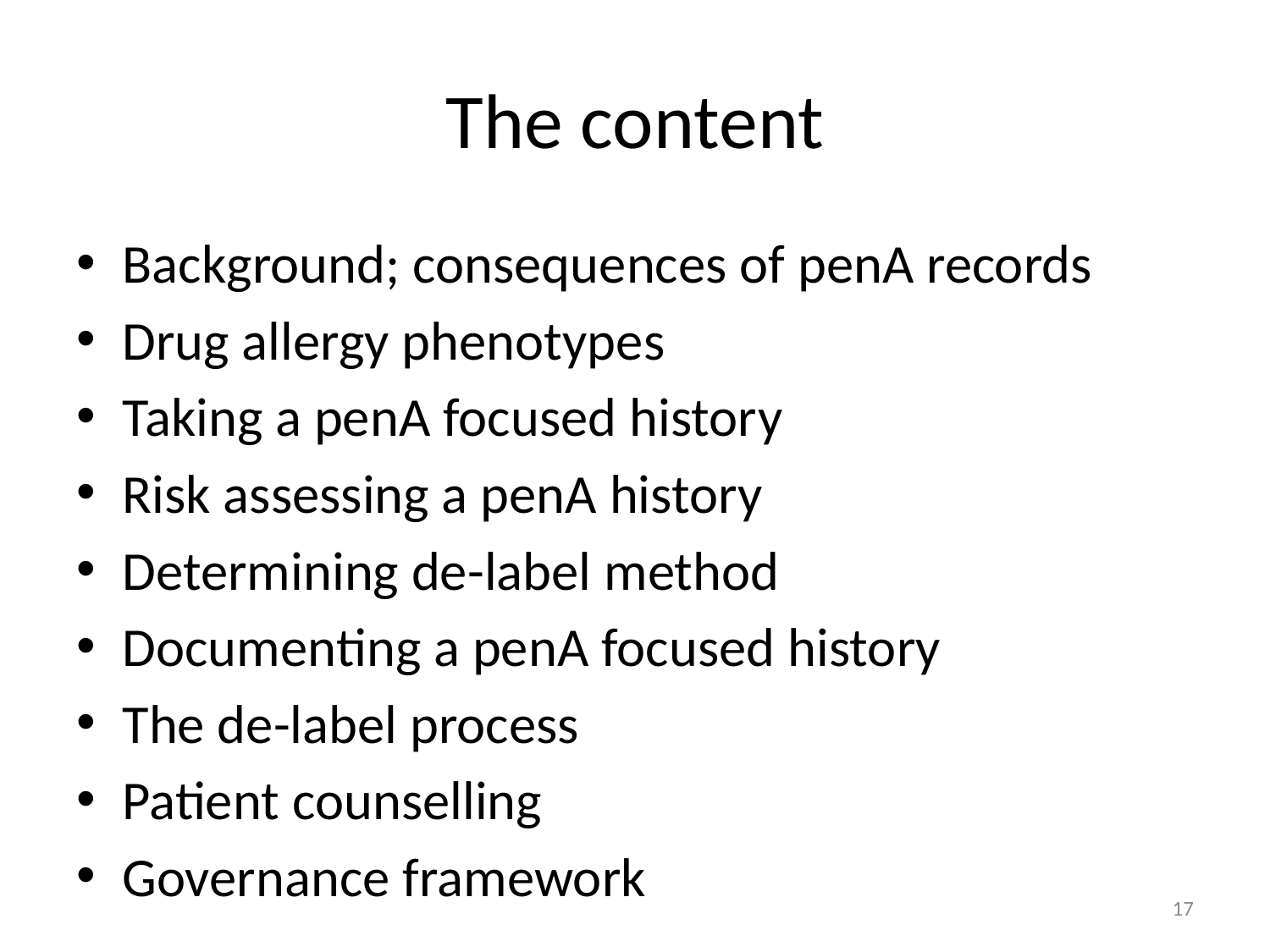

# The content
Background; consequences of penA records
Drug allergy phenotypes
Taking a penA focused history
Risk assessing a penA history
Determining de-label method
Documenting a penA focused history
The de-label process
Patient counselling
Governance framework
17

## Slide 18
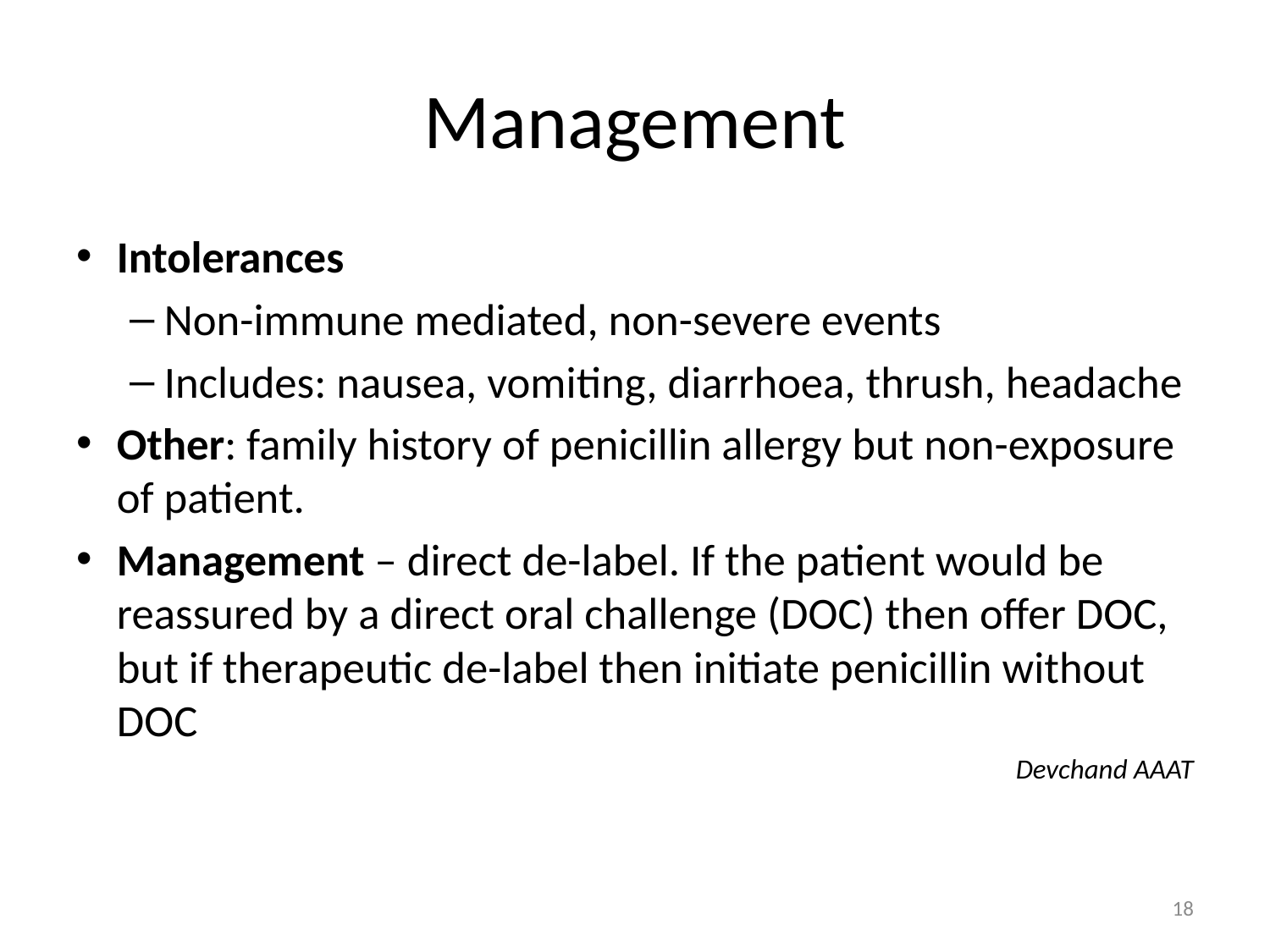

# Management
Intolerances
Non-immune mediated, non-severe events
Includes: nausea, vomiting, diarrhoea, thrush, headache
Other: family history of penicillin allergy but non-exposure of patient.
Management – direct de-label. If the patient would be reassured by a direct oral challenge (DOC) then offer DOC, but if therapeutic de-label then initiate penicillin without DOC
Devchand AAAT
18

## Slide 19
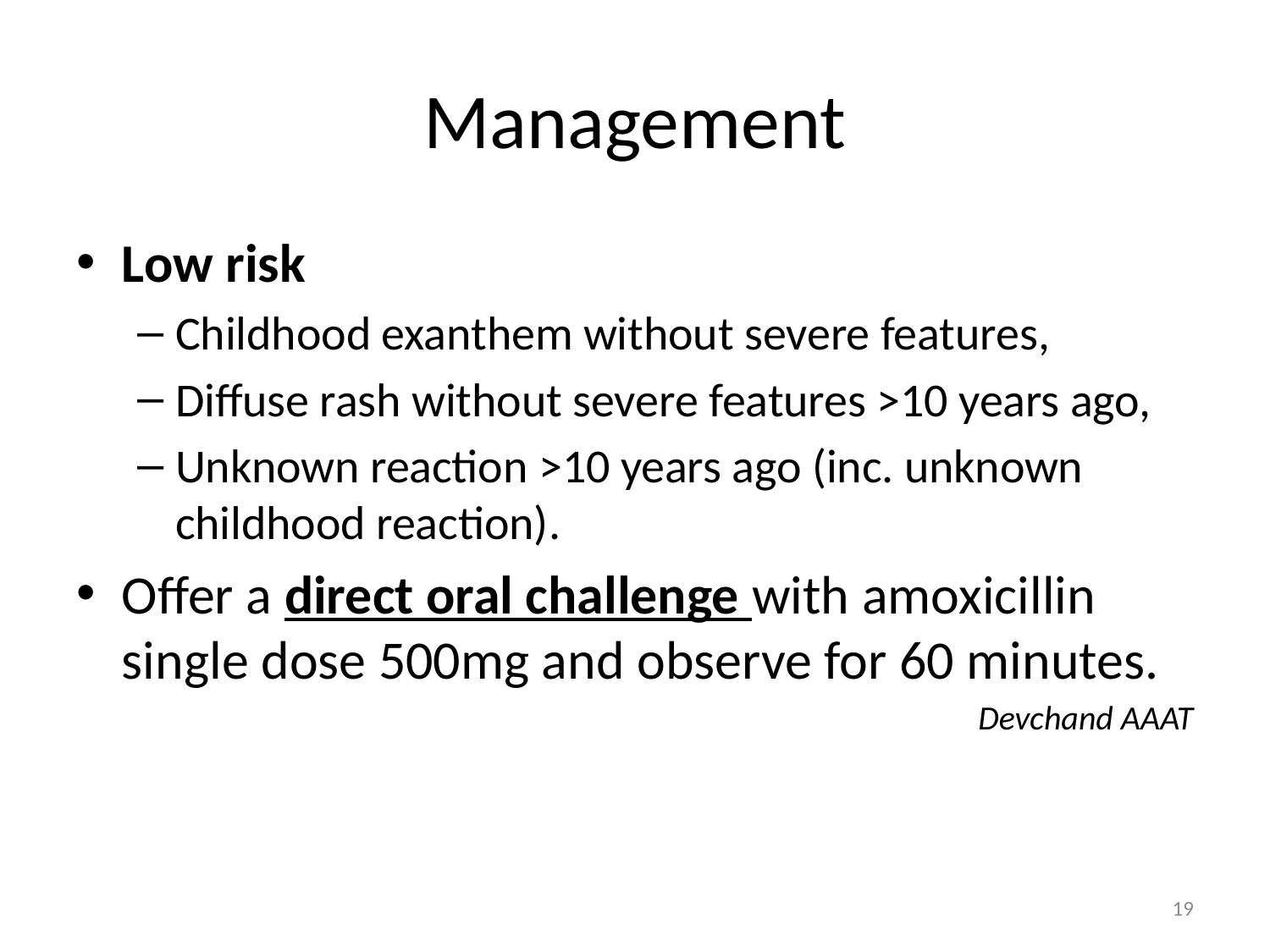

# Management
Low risk
Childhood exanthem without severe features,
Diffuse rash without severe features >10 years ago,
Unknown reaction >10 years ago (inc. unknown childhood reaction).
Offer a direct oral challenge with amoxicillin single dose 500mg and observe for 60 minutes.
Devchand AAAT
19

## Slide 20
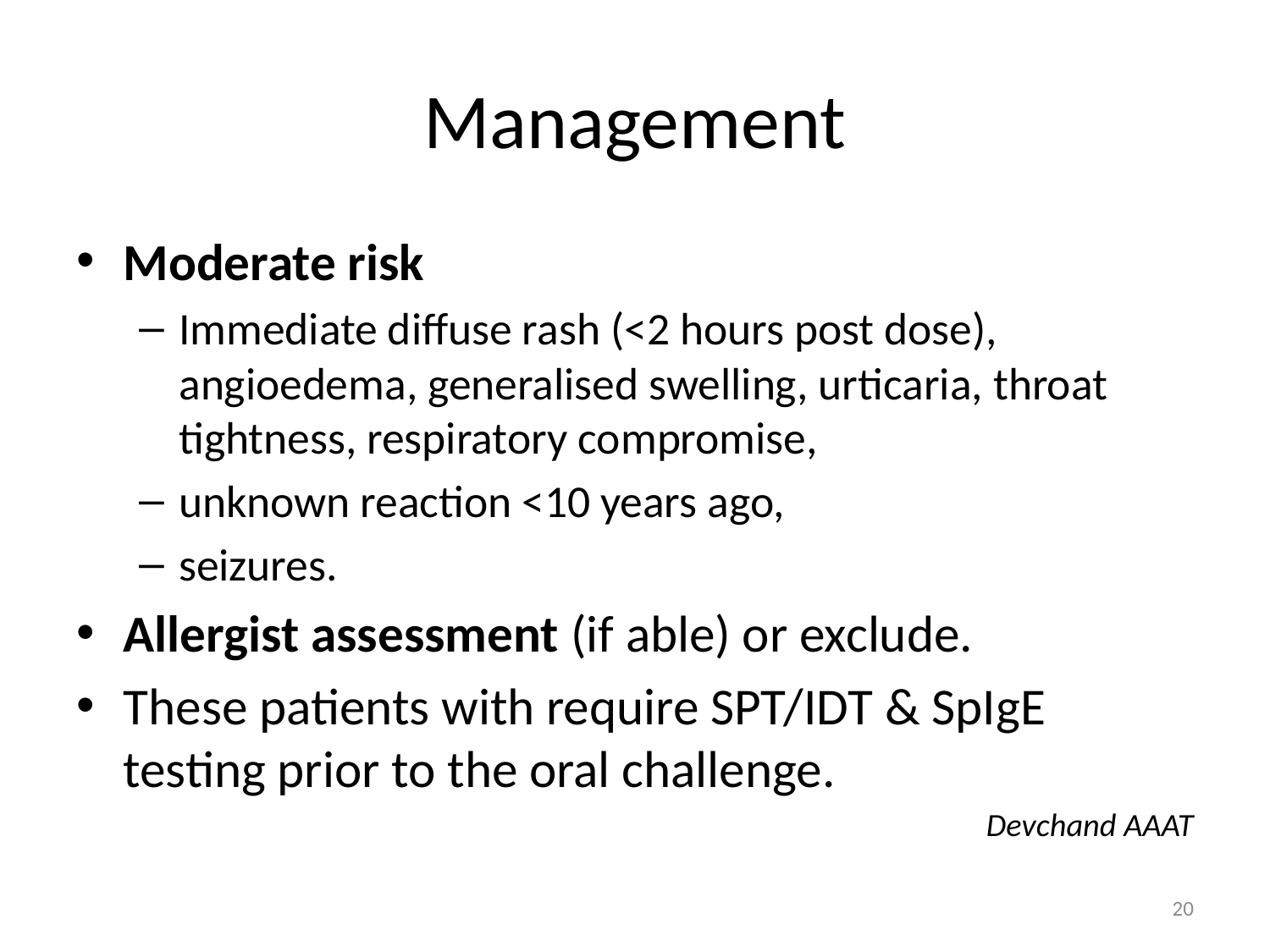

# Management
Moderate risk
Immediate diffuse rash (<2 hours post dose), angioedema, generalised swelling, urticaria, throat tightness, respiratory compromise,
unknown reaction <10 years ago,
seizures.
Allergist assessment (if able) or exclude.
These patients with require SPT/IDT & SpIgE testing prior to the oral challenge.
Devchand AAAT
20

## Slide 21
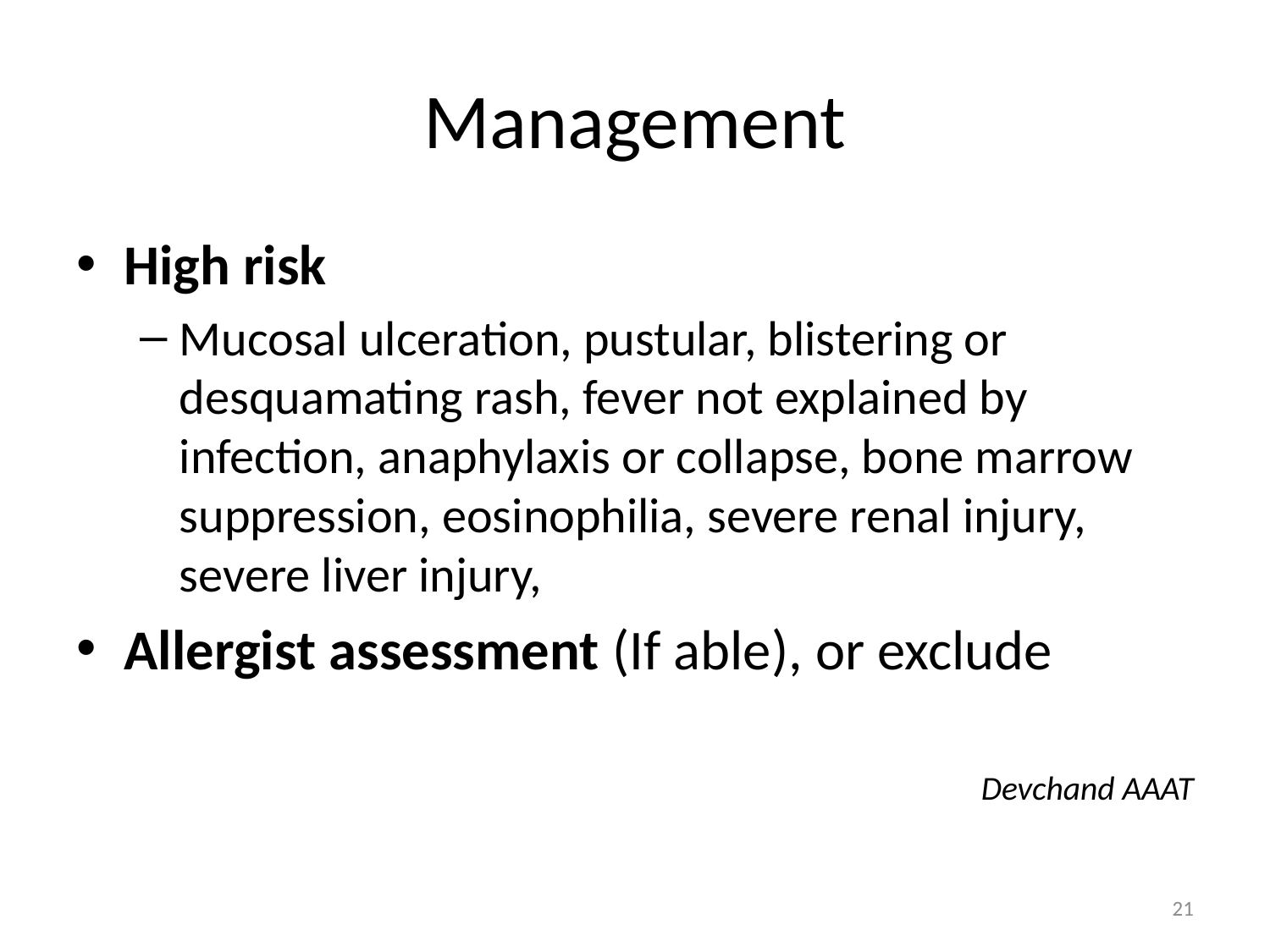

# Management
High risk
Mucosal ulceration, pustular, blistering or desquamating rash, fever not explained by infection, anaphylaxis or collapse, bone marrow suppression, eosinophilia, severe renal injury, severe liver injury,
Allergist assessment (If able), or exclude
Devchand AAAT
21

## Slide 22
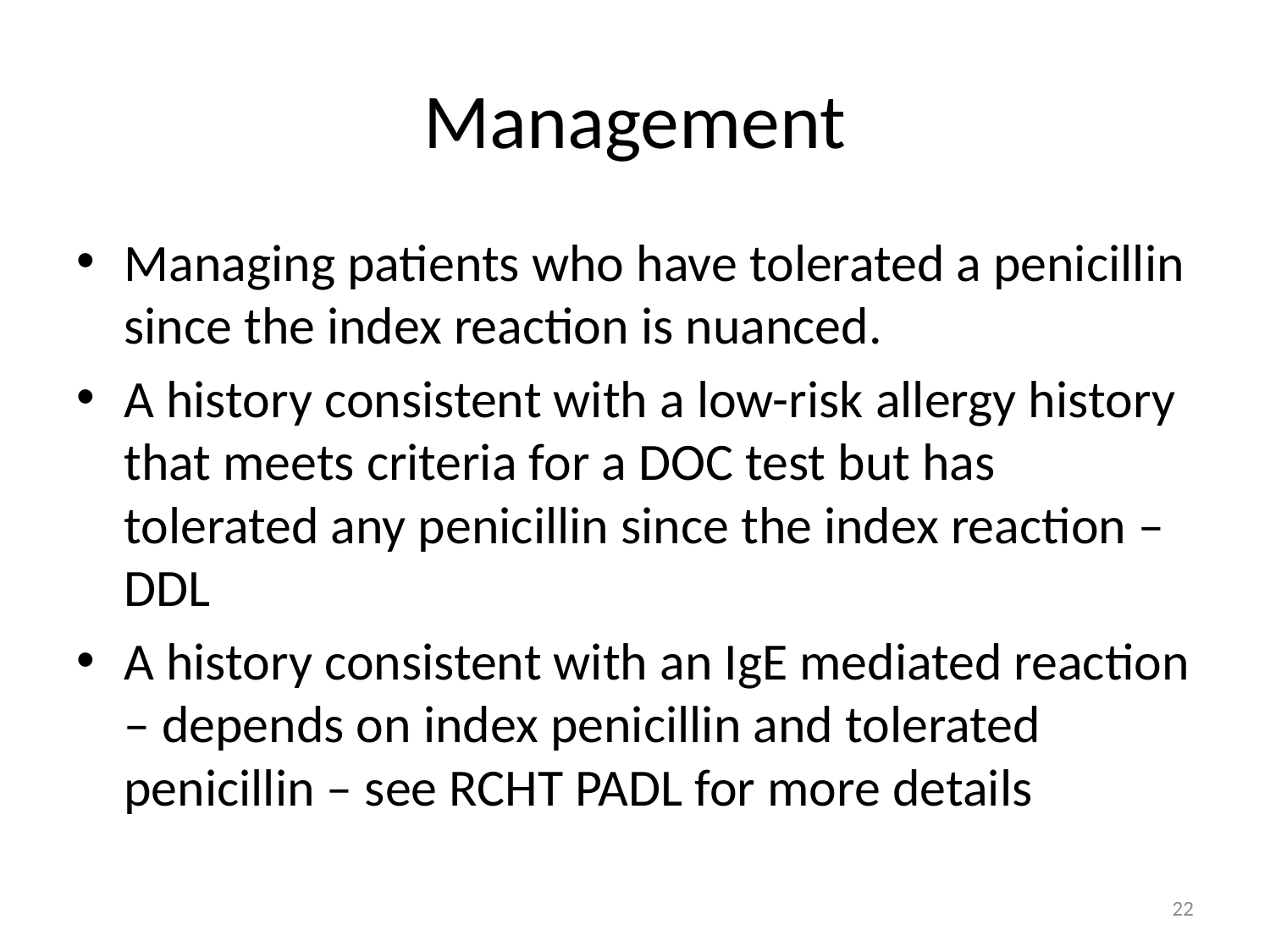

# Management
Managing patients who have tolerated a penicillin since the index reaction is nuanced.
A history consistent with a low-risk allergy history that meets criteria for a DOC test but has tolerated any penicillin since the index reaction – DDL
A history consistent with an IgE mediated reaction – depends on index penicillin and tolerated penicillin – see RCHT PADL for more details
22

## Slide 23
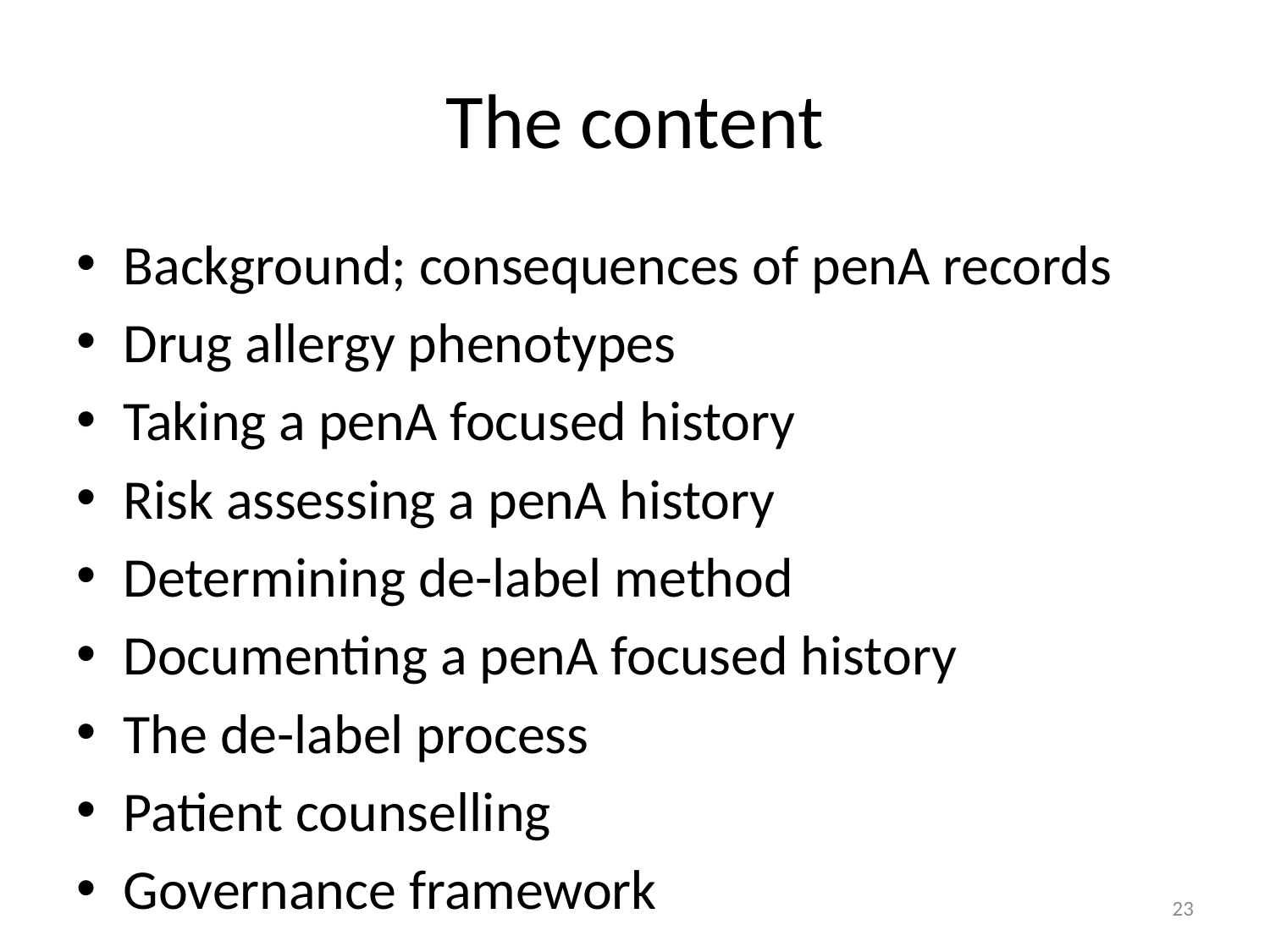

# The content
Background; consequences of penA records
Drug allergy phenotypes
Taking a penA focused history
Risk assessing a penA history
Determining de-label method
Documenting a penA focused history
The de-label process
Patient counselling
Governance framework
23

## Slide 24
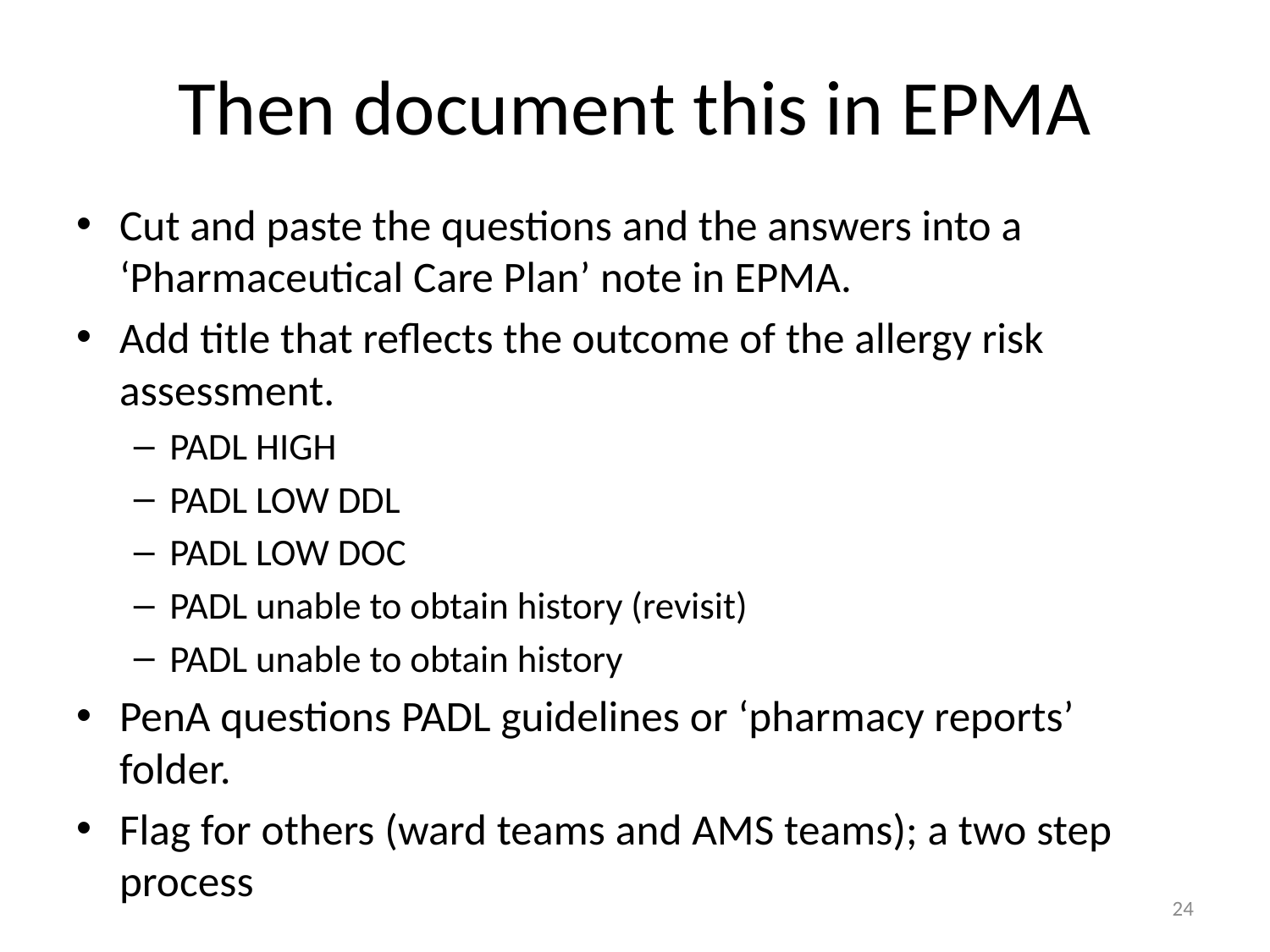

# Then document this in EPMA
Cut and paste the questions and the answers into a ‘Pharmaceutical Care Plan’ note in EPMA.
Add title that reflects the outcome of the allergy risk assessment.
PADL HIGH
PADL LOW DDL
PADL LOW DOC
PADL unable to obtain history (revisit)
PADL unable to obtain history
PenA questions PADL guidelines or ‘pharmacy reports’ folder.
Flag for others (ward teams and AMS teams); a two step process
24

## Slide 25
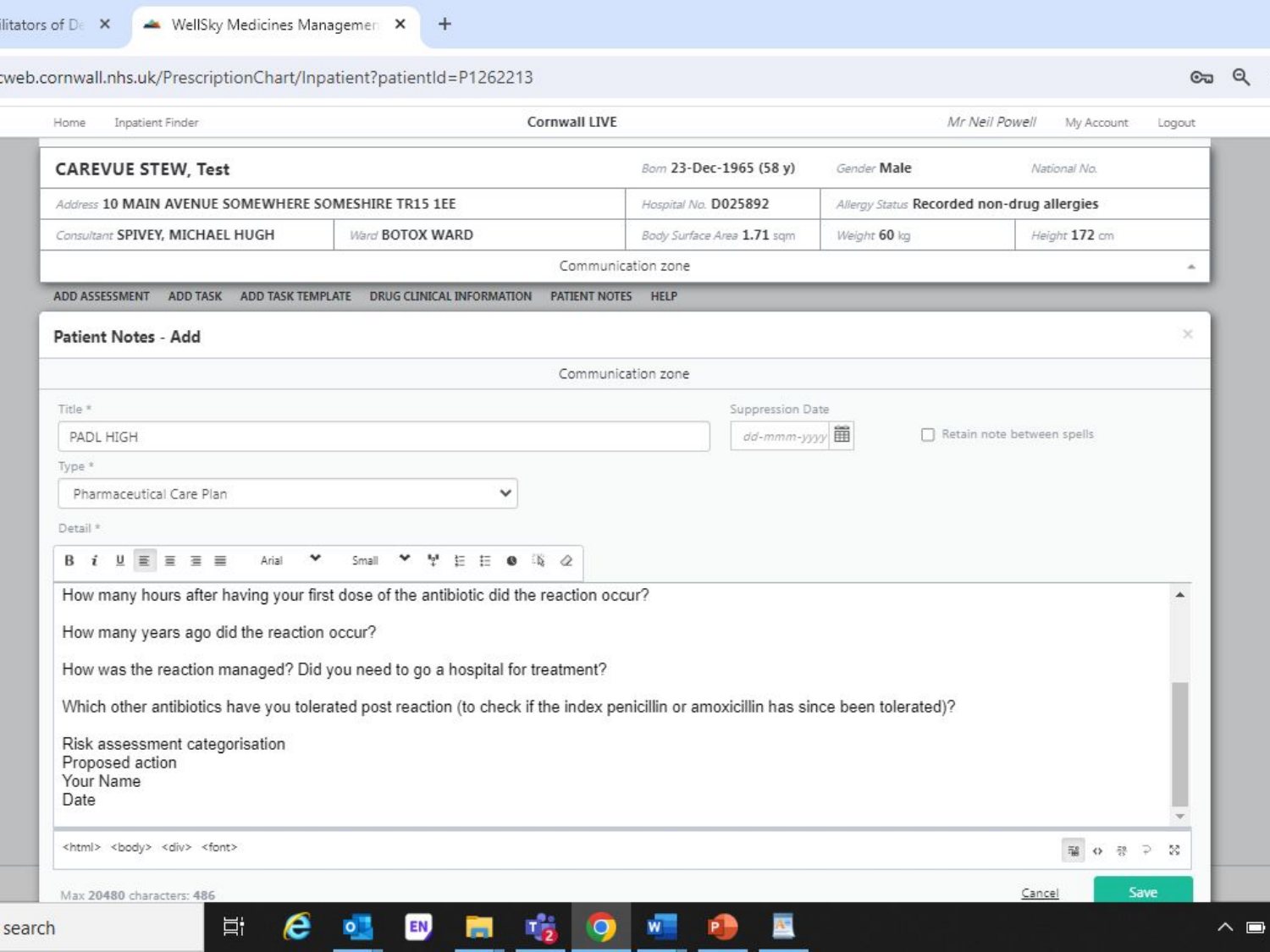

25

## Slide 26
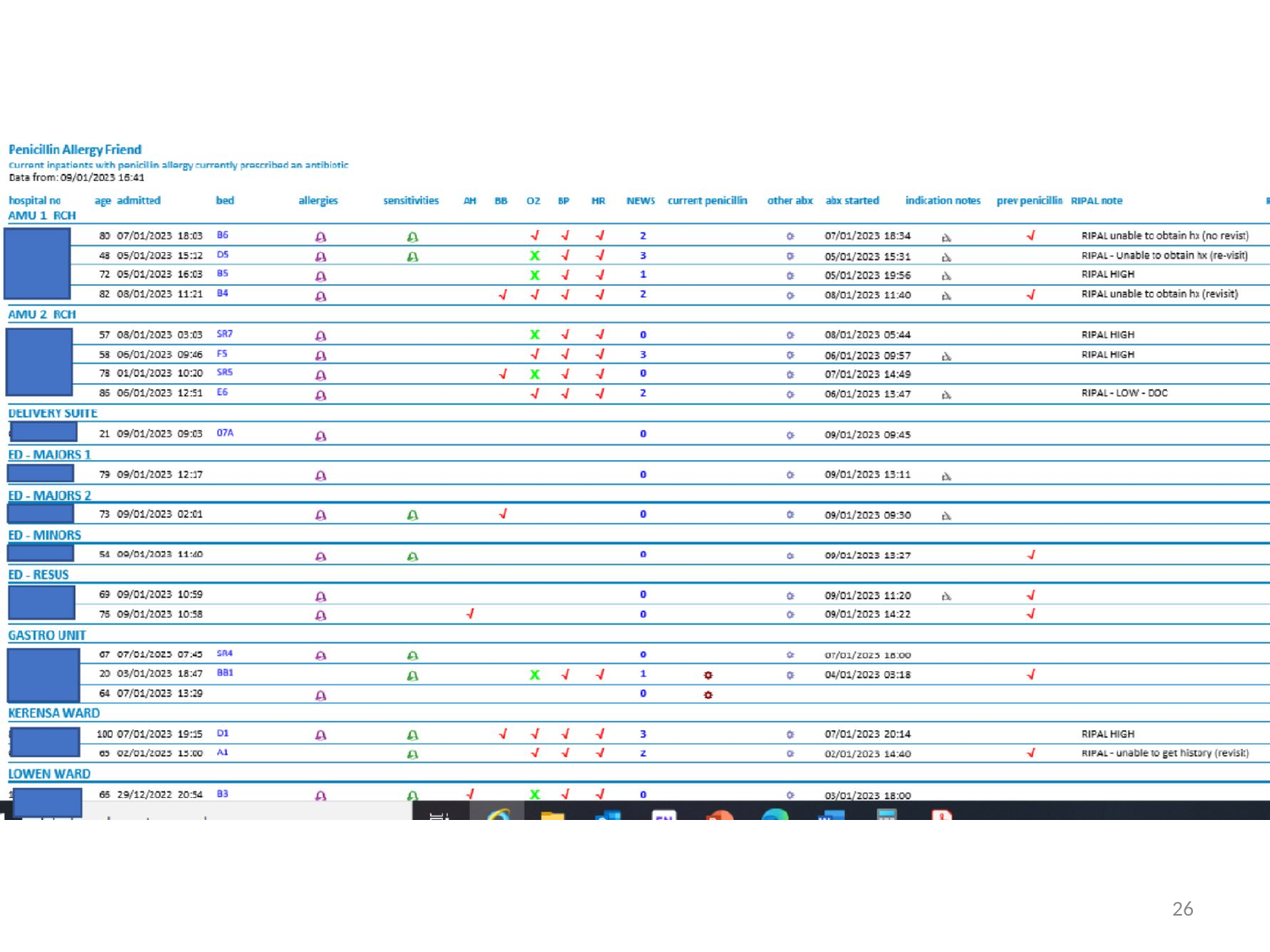

26

## Slide 27
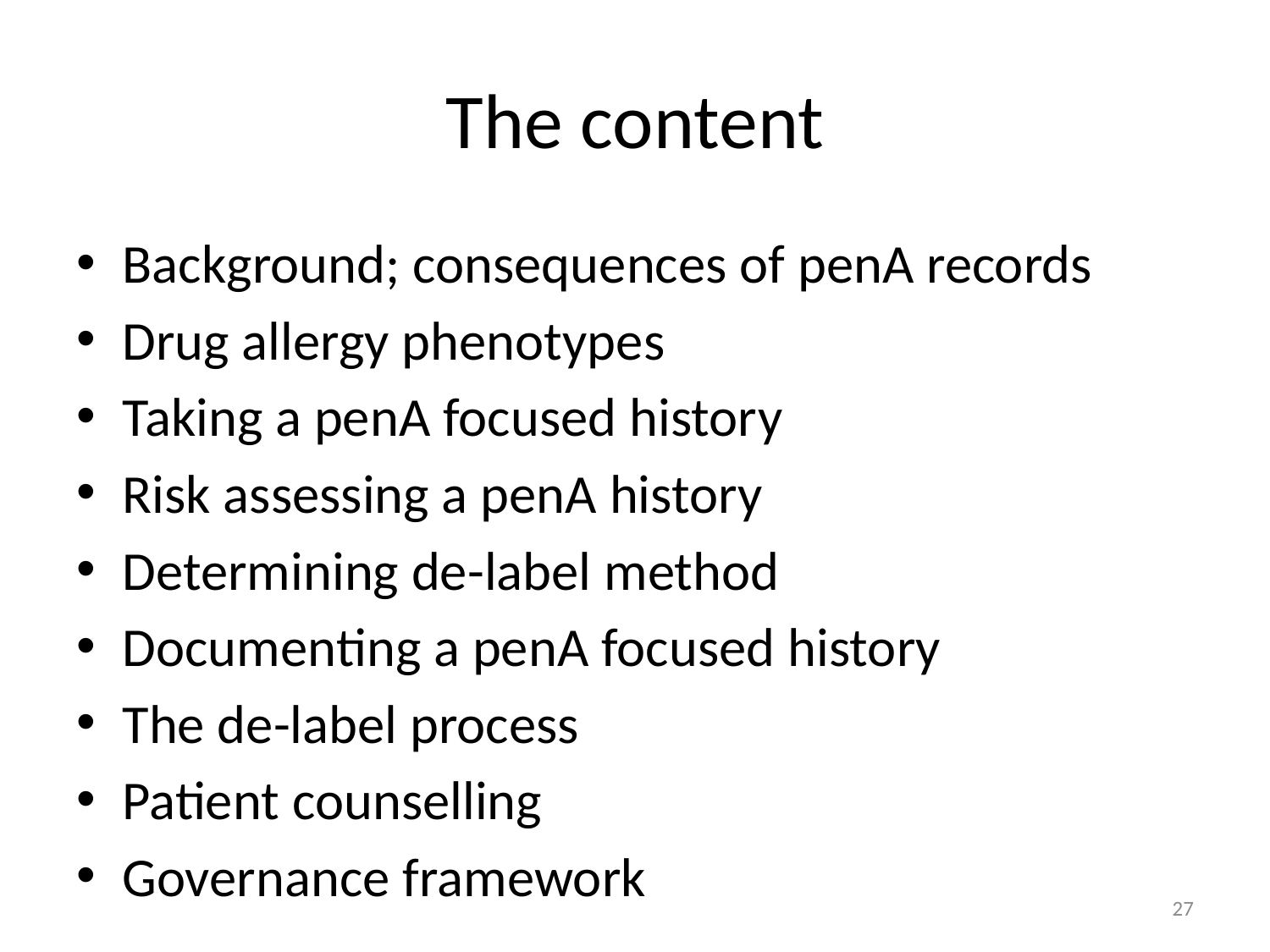

# The content
Background; consequences of penA records
Drug allergy phenotypes
Taking a penA focused history
Risk assessing a penA history
Determining de-label method
Documenting a penA focused history
The de-label process
Patient counselling
Governance framework
27

## Slide 28
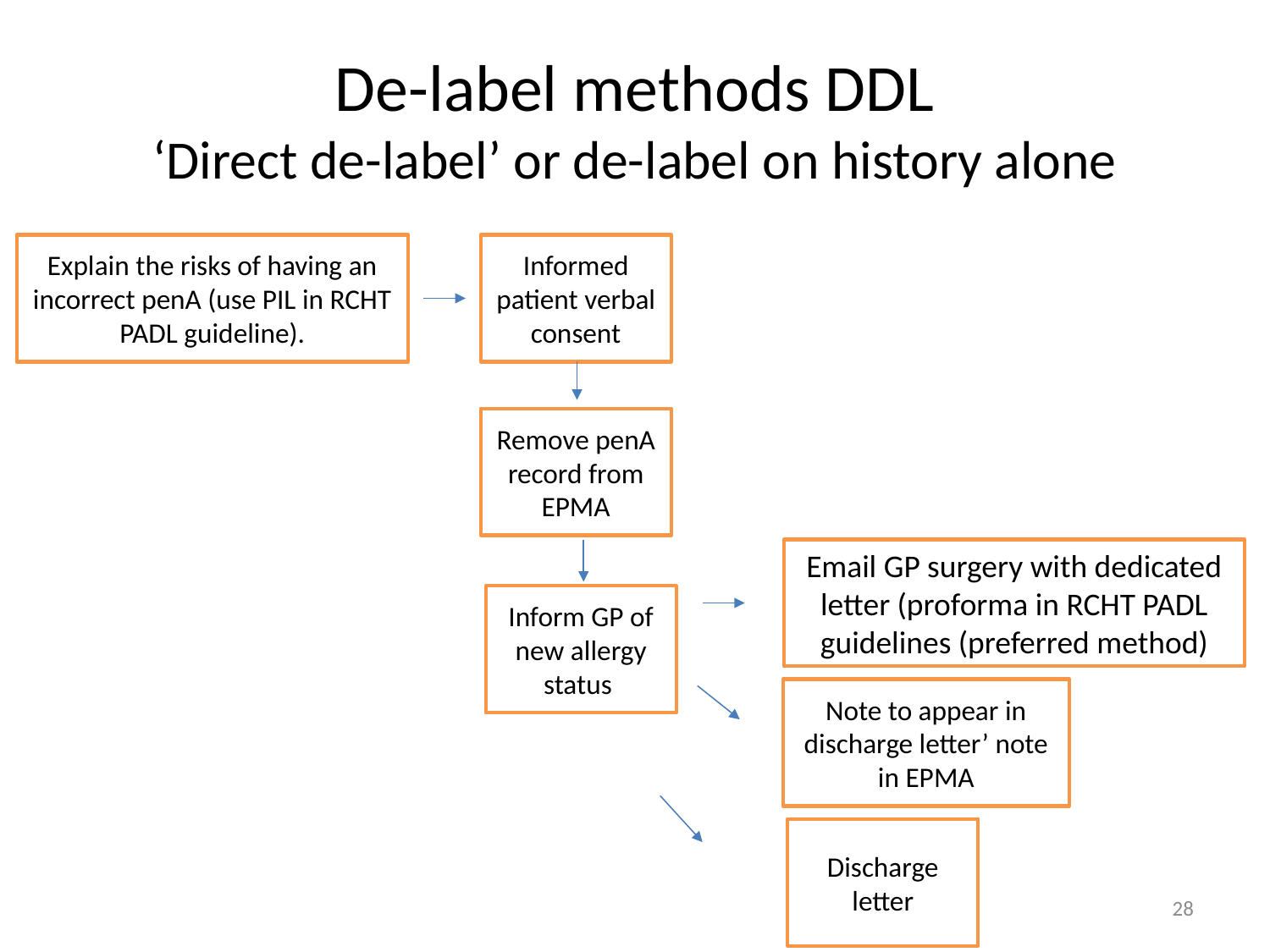

# De-label methods DDL ‘Direct de-label’ or de-label on history alone
Explain the risks of having an incorrect penA (use PIL in RCHT PADL guideline).
Informed patient verbal consent
Remove penA record from EPMA
Email GP surgery with dedicated letter (proforma in RCHT PADL guidelines (preferred method)
Inform GP of new allergy status
Note to appear in discharge letter’ note in EPMA
Discharge letter
28

## Slide 29
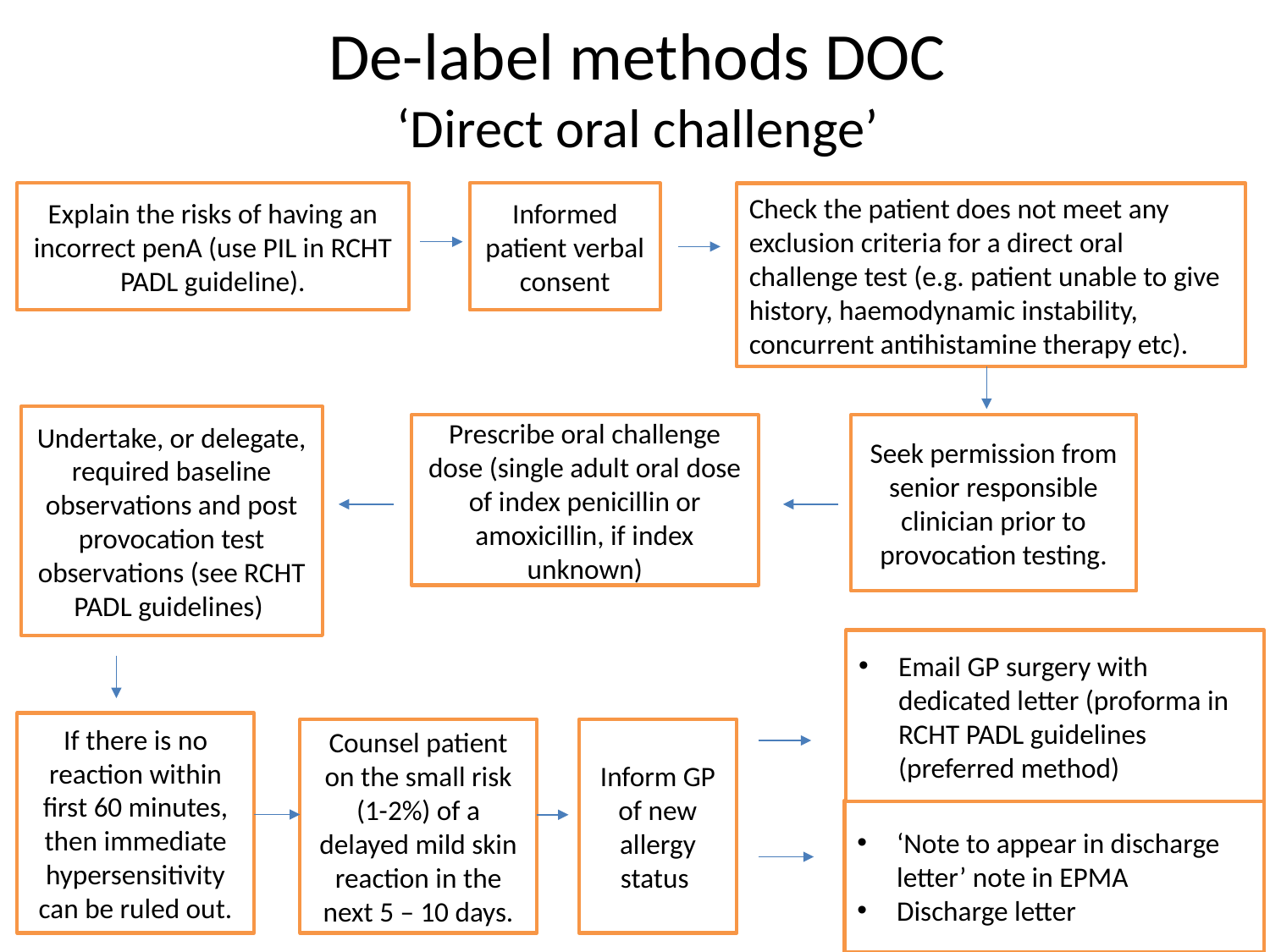

# De-label methods DOC ‘Direct oral challenge’
Explain the risks of having an incorrect penA (use PIL in RCHT PADL guideline).
Informed patient verbal consent
Check the patient does not meet any exclusion criteria for a direct oral challenge test (e.g. patient unable to give history, haemodynamic instability, concurrent antihistamine therapy etc).
Undertake, or delegate, required baseline observations and post provocation test observations (see RCHT PADL guidelines)
Prescribe oral challenge dose (single adult oral dose of index penicillin or amoxicillin, if index unknown)
Seek permission from senior responsible clinician prior to provocation testing.
Email GP surgery with dedicated letter (proforma in RCHT PADL guidelines (preferred method)
If there is no reaction within first 60 minutes, then immediate hypersensitivity can be ruled out.
Counsel patient on the small risk (1-2%) of a delayed mild skin reaction in the next 5 – 10 days.
Inform GP of new allergy status
‘Note to appear in discharge letter’ note in EPMA
Discharge letter
29

## Slide 30
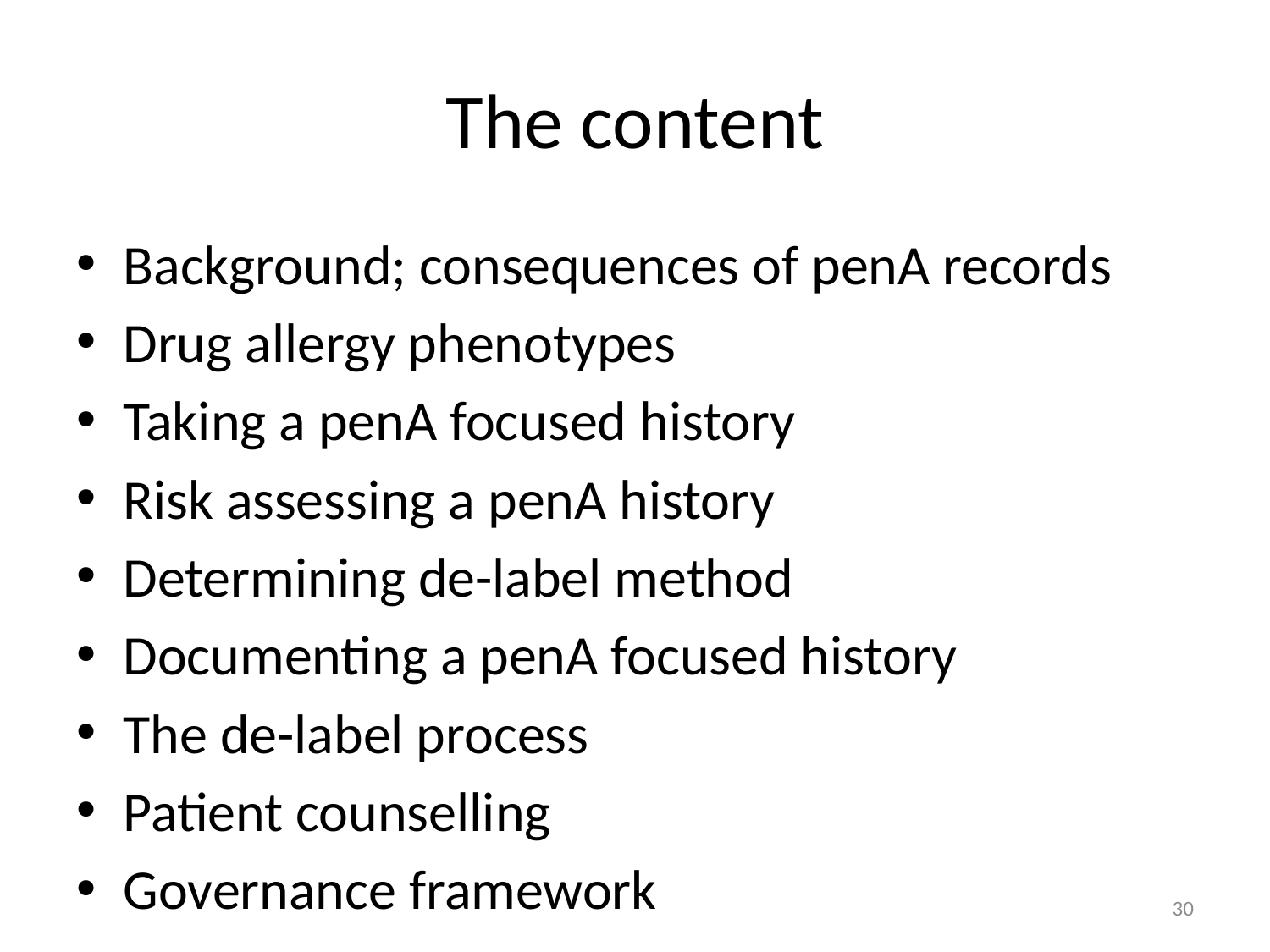

# The content
Background; consequences of penA records
Drug allergy phenotypes
Taking a penA focused history
Risk assessing a penA history
Determining de-label method
Documenting a penA focused history
The de-label process
Patient counselling
Governance framework
30

## Slide 31
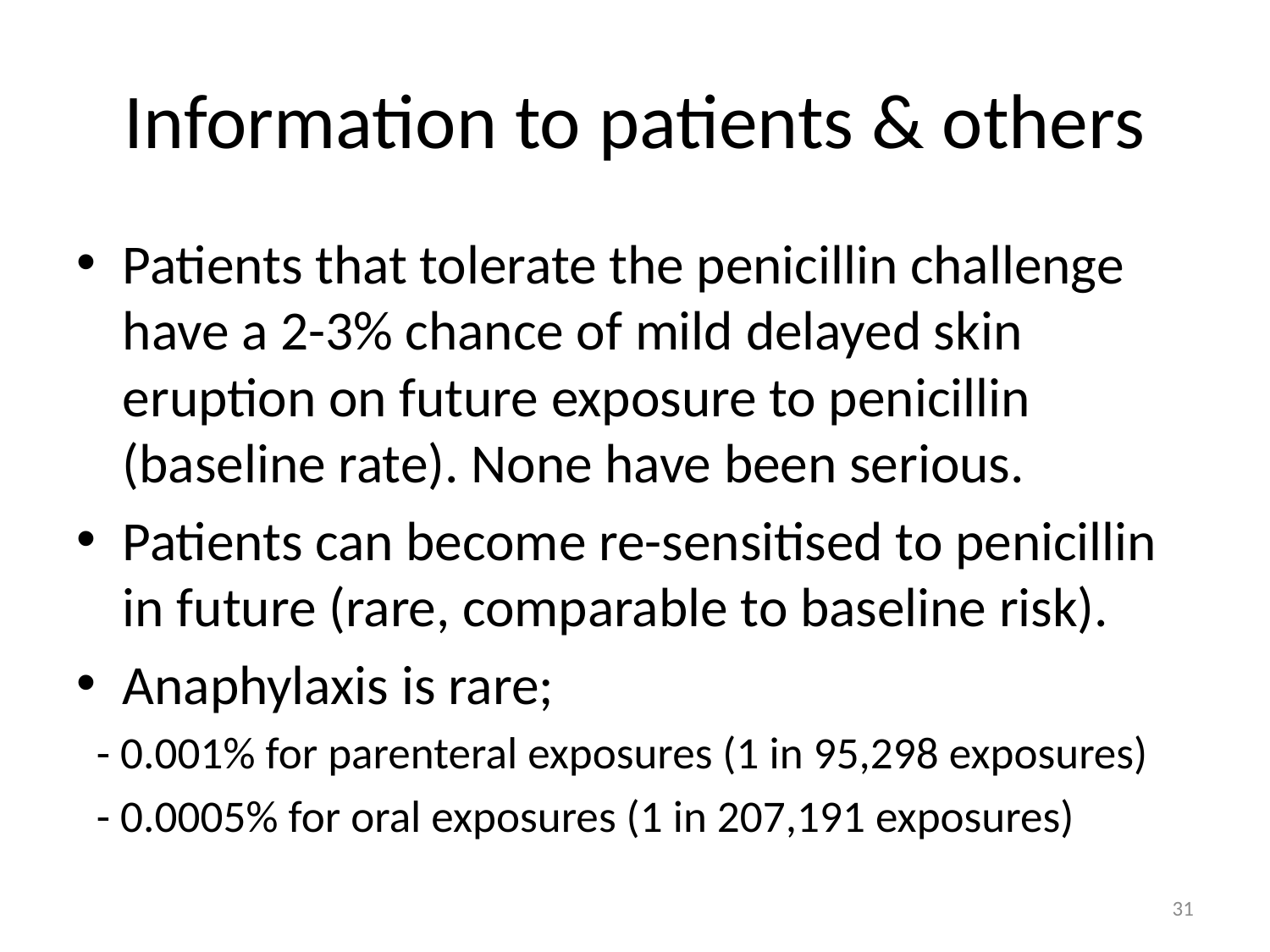

# Information to patients & others
Patients that tolerate the penicillin challenge have a 2-3% chance of mild delayed skin eruption on future exposure to penicillin (baseline rate). None have been serious.
Patients can become re-sensitised to penicillin in future (rare, comparable to baseline risk).
Anaphylaxis is rare;
 - 0.001% for parenteral exposures (1 in 95,298 exposures)
 - 0.0005% for oral exposures (1 in 207,191 exposures)
31

## Slide 32
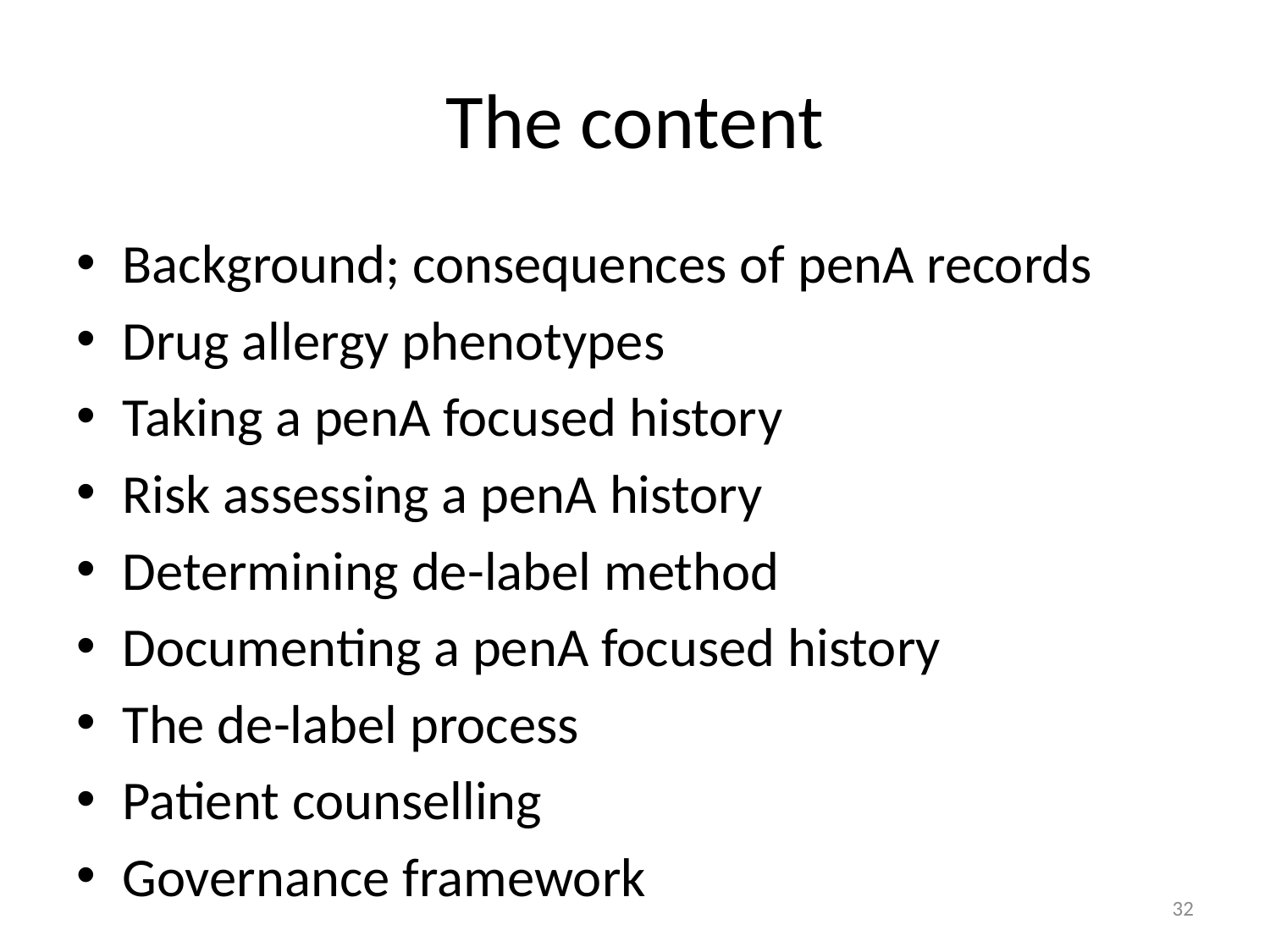

# The content
Background; consequences of penA records
Drug allergy phenotypes
Taking a penA focused history
Risk assessing a penA history
Determining de-label method
Documenting a penA focused history
The de-label process
Patient counselling
Governance framework
32

## Slide 33
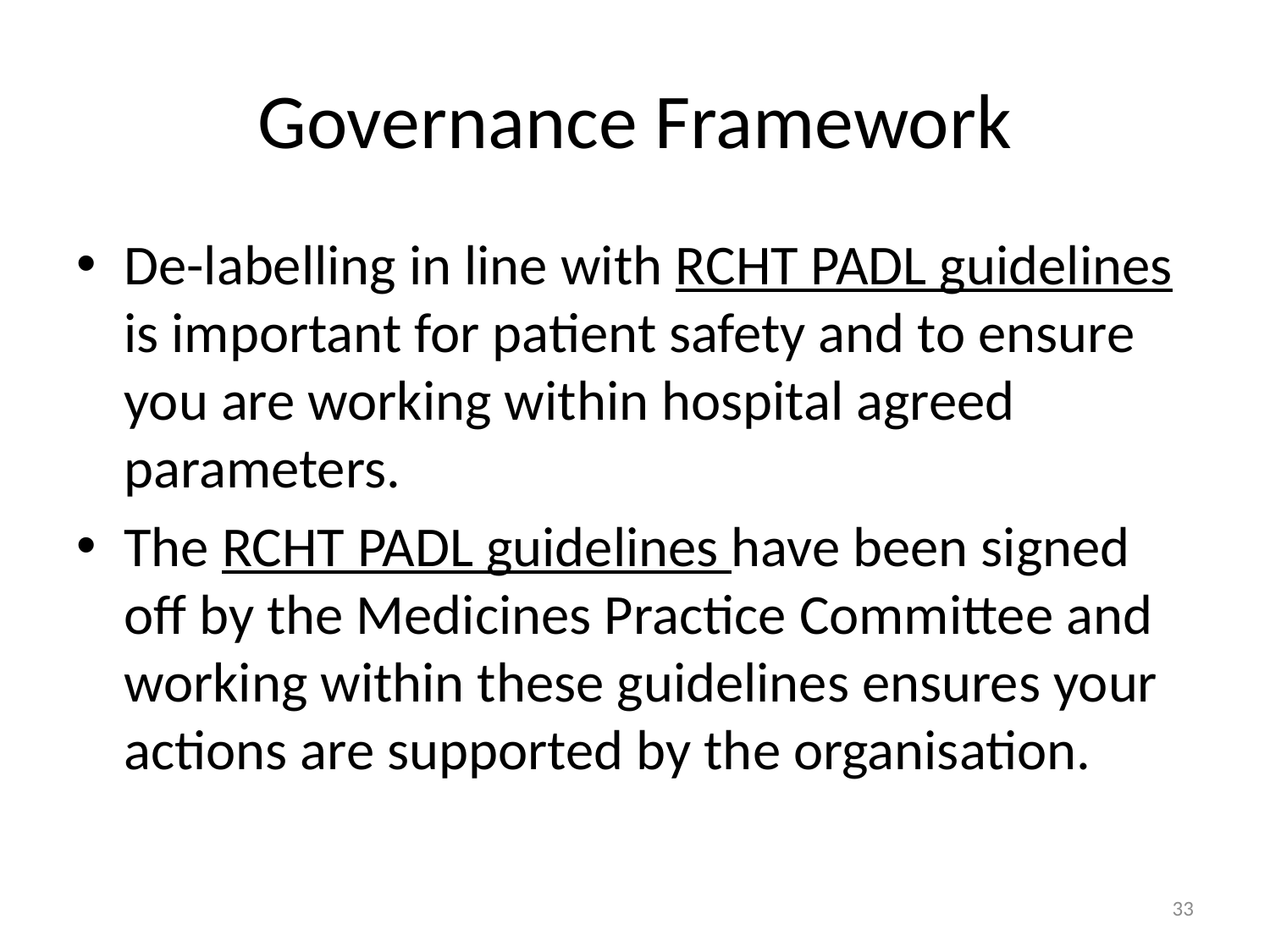

# Governance Framework
De-labelling in line with RCHT PADL guidelines is important for patient safety and to ensure you are working within hospital agreed parameters.
The RCHT PADL guidelines have been signed off by the Medicines Practice Committee and working within these guidelines ensures your actions are supported by the organisation.
33

## Slide 34
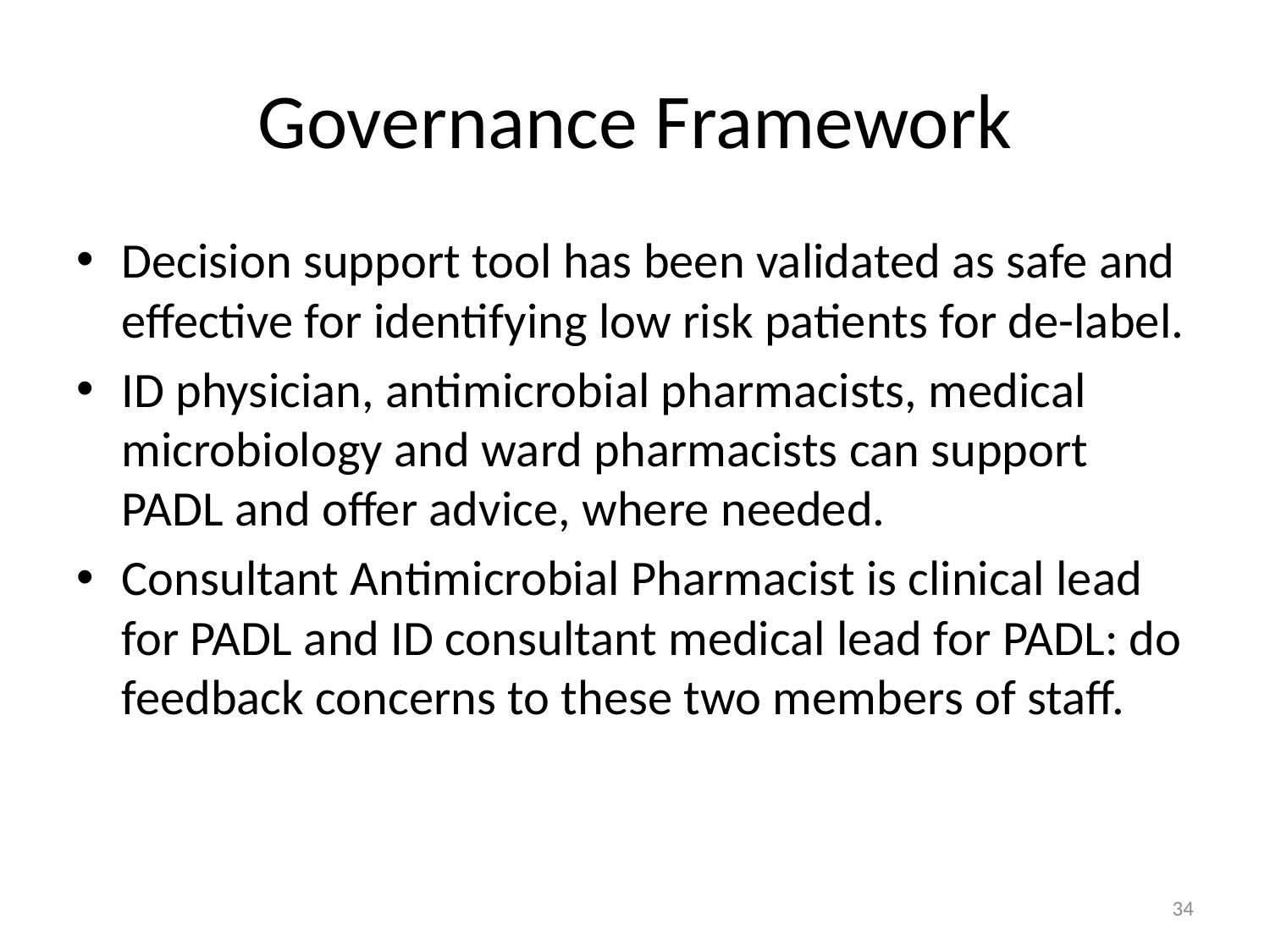

# Governance Framework
Decision support tool has been validated as safe and effective for identifying low risk patients for de-label.
ID physician, antimicrobial pharmacists, medical microbiology and ward pharmacists can support PADL and offer advice, where needed.
Consultant Antimicrobial Pharmacist is clinical lead for PADL and ID consultant medical lead for PADL: do feedback concerns to these two members of staff.
34

## Slide 35
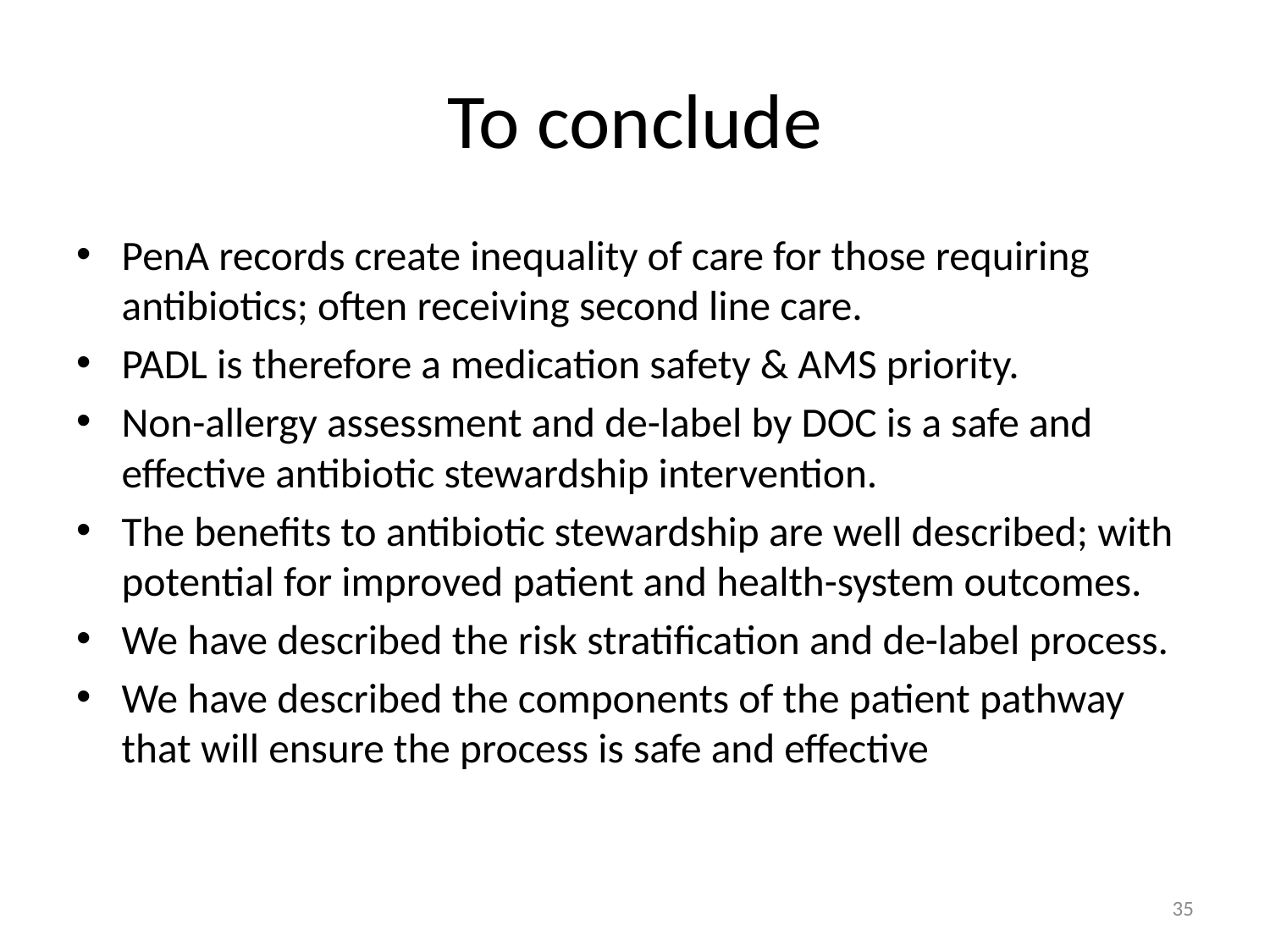

# To conclude
PenA records create inequality of care for those requiring antibiotics; often receiving second line care.
PADL is therefore a medication safety & AMS priority.
Non-allergy assessment and de-label by DOC is a safe and effective antibiotic stewardship intervention.
The benefits to antibiotic stewardship are well described; with potential for improved patient and health-system outcomes.
We have described the risk stratification and de-label process.
We have described the components of the patient pathway that will ensure the process is safe and effective
35

## Slide 36
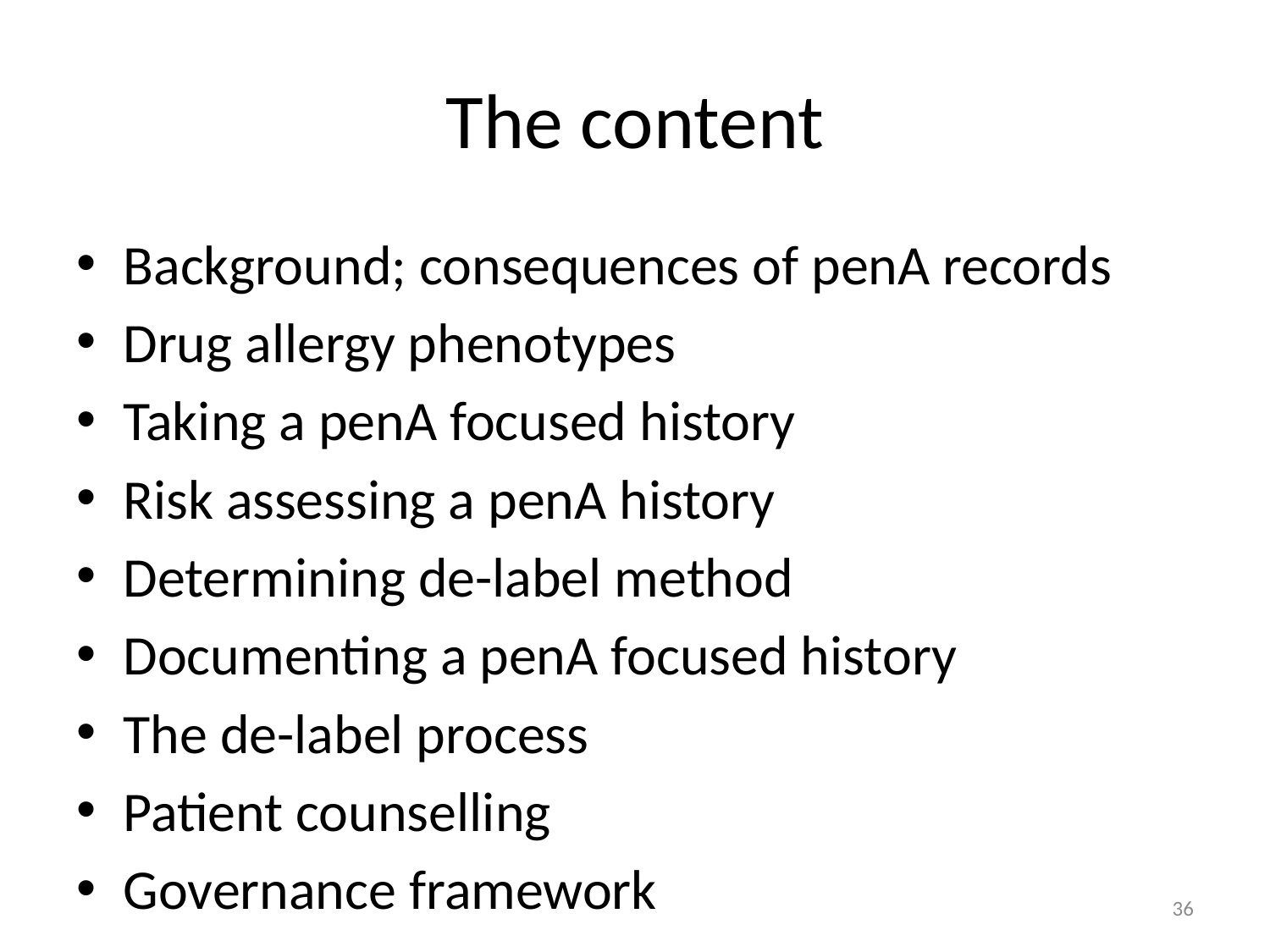

# The content
Background; consequences of penA records
Drug allergy phenotypes
Taking a penA focused history
Risk assessing a penA history
Determining de-label method
Documenting a penA focused history
The de-label process
Patient counselling
Governance framework
36

## Slide 37
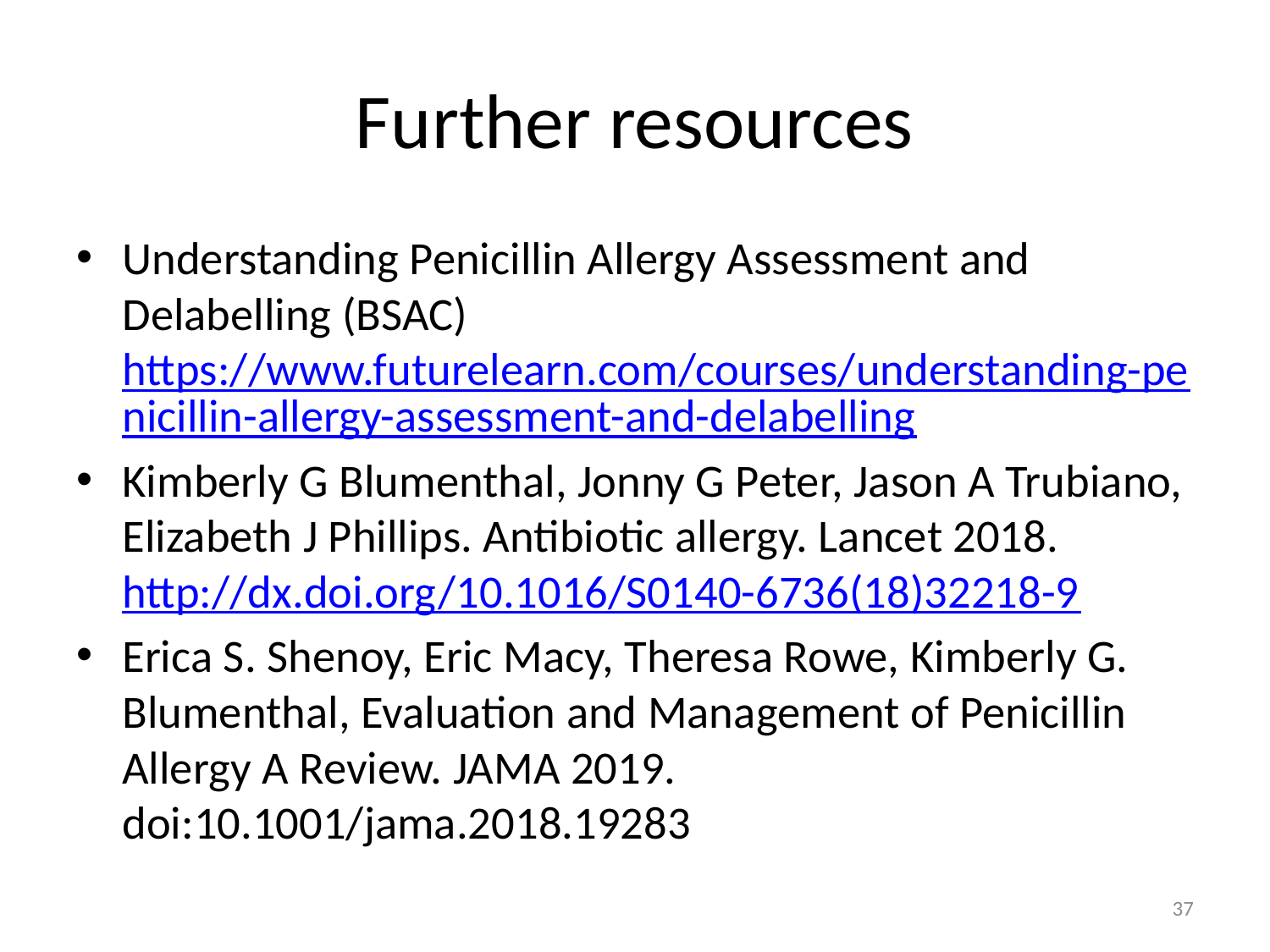

# Further resources
Understanding Penicillin Allergy Assessment and Delabelling (BSAC) https://www.futurelearn.com/courses/understanding-penicillin-allergy-assessment-and-delabelling
Kimberly G Blumenthal, Jonny G Peter, Jason A Trubiano, Elizabeth J Phillips. Antibiotic allergy. Lancet 2018. http://dx.doi.org/10.1016/S0140-6736(18)32218-9
Erica S. Shenoy, Eric Macy, Theresa Rowe, Kimberly G. Blumenthal, Evaluation and Management of Penicillin Allergy A Review. JAMA 2019. doi:10.1001/jama.2018.19283
37

## Slide 38
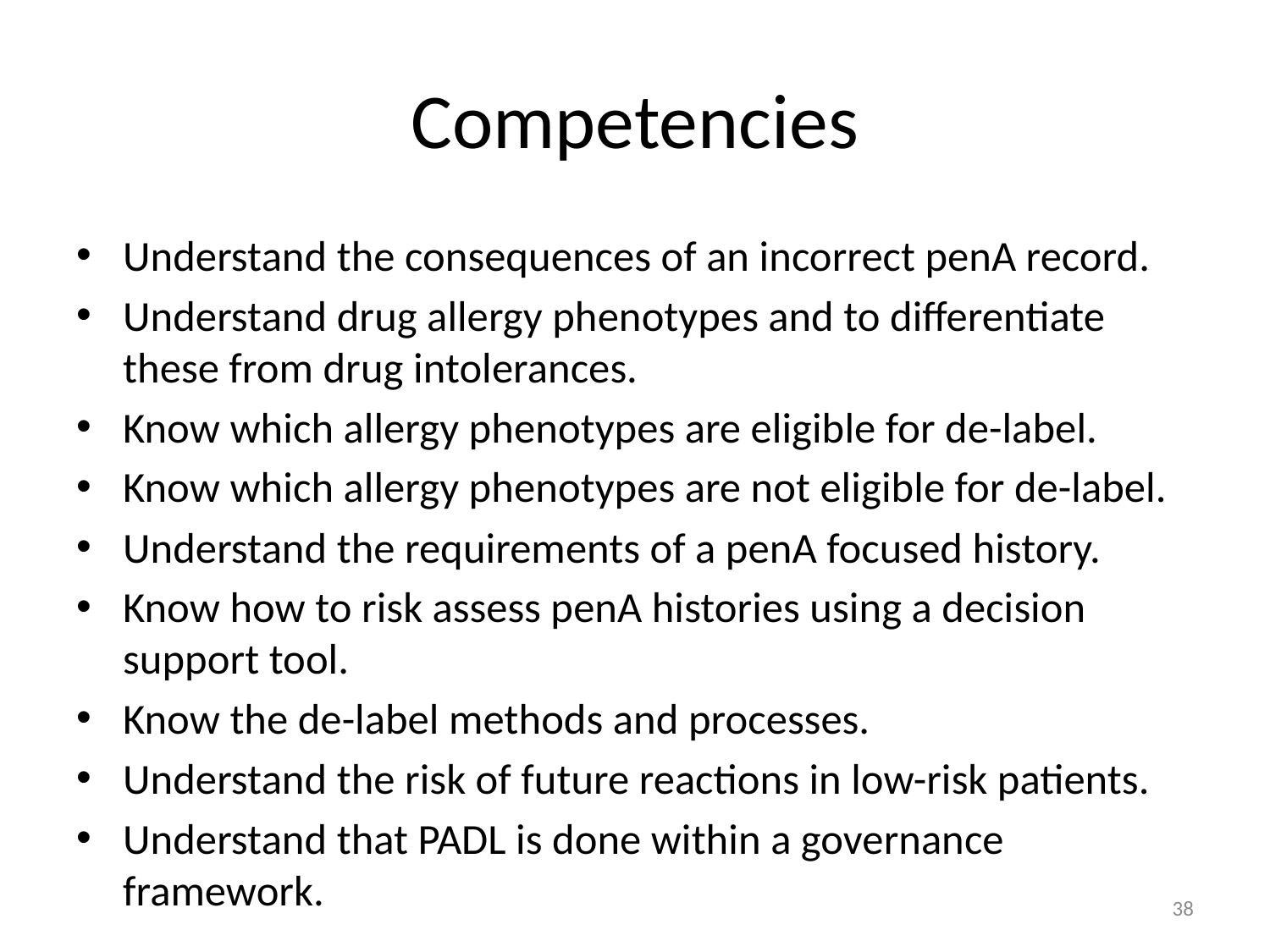

# Competencies
Understand the consequences of an incorrect penA record.
Understand drug allergy phenotypes and to differentiate these from drug intolerances.
Know which allergy phenotypes are eligible for de-label.
Know which allergy phenotypes are not eligible for de-label.
Understand the requirements of a penA focused history.
Know how to risk assess penA histories using a decision support tool.
Know the de-label methods and processes.
Understand the risk of future reactions in low-risk patients.
Understand that PADL is done within a governance framework.
38
