# Supplementary material for: A nurse-run, pharmacist-led outpatient penicillin allergy de-label clinic in the UK
Source: JAC Antimicrob Resist. 2026 Feb 2;8(1):dlag005. doi: 10.1093/jacamr/dlag005 (PMC12862639; doi:10.1093/jacamr/dlag005)
Supplement: dlag005_Supplementary_Data [file dlag005_supplementary_data.zip › Penicillin Allergy De-Labelling in Adult Inpatients Clinical Guideline V1.2.docx]

**Penicillin Allergy De-Labelling in Adult Inpatients Clinical Guideline**

**V1.1**

**June 2024**

**Summary**


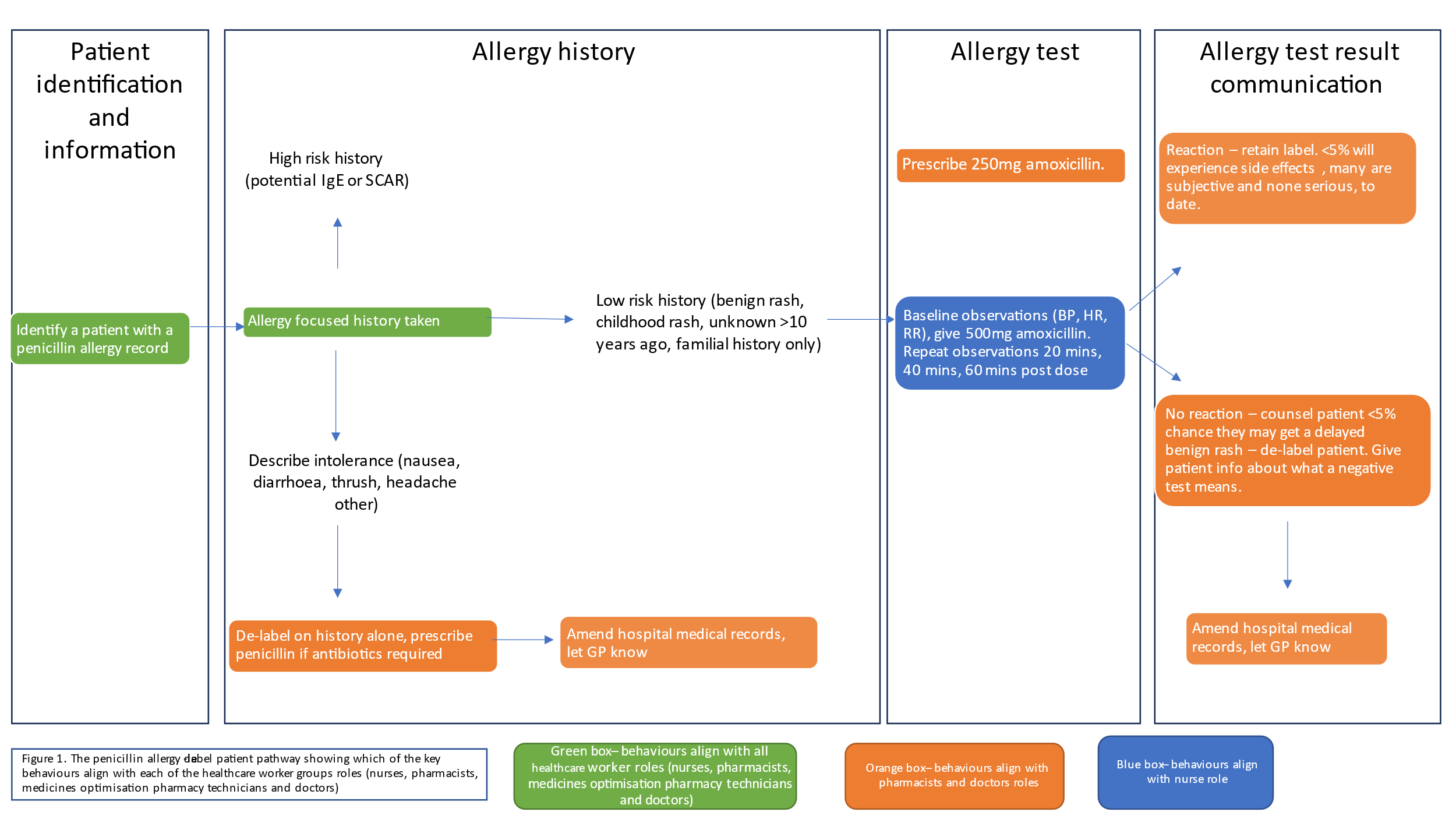


## Aim/Purpose of this Guideline

- 1. The purpose of this guideline is to support safe penicillin allergy de-labelling and optimise antibiotic treatment.
  2. Removing inappropriate penicillin allergy records reduces unnecessary second and third-line antibiotics and reduces broad spectrum antibiotic usage in hospitalised patients.
  3. Most patients with a penicillin allergy label (>95%) do not have a true penicillin allergy and can take penicillin safely.
  4. This guideline is designed to support and facilitate penicillin allergy risk assessment, to assess whether patients can be de-labelled safely.
  5. This version supersedes any previous versions of this document.

**Data Protection Act 2018 (UK General Data Protection Regulation – GDPR) Legislation**

The Trust has a duty under the Data Protection Act 2018 and UK General Data Protection Regulations 2016/679 to ensure that there is a valid legal basis to process personal and sensitive data. The legal basis for processing must be identified and documented before the processing begins. In many cases we may need consent; this must be explicit, informed, and documented. We cannot rely on opt out, it must be opt in.

Data Protection Act 2018 and UK General Data Protection Regulations 2016/679 is applicable to all staff; this includes those working as contractors and providers of services.

For more information about your obligations under the Data Protection Act 2018 and UK General Data Protection Regulations 2016/679 please see the Information Use Framework Policy or contact the Information Governance Team

Royal Cornwall Hospital Trust [rch-tr.infogov@nhs.net](mailto:rch-tr.infogov@nhs.net)

## The Guidance

- 1. **Allergy assessment**
     1. Adult patients (>18 years of age) admitted to hospital with a penicillin allergy record preventing the use of a penicillin antibiotic and have a low-risk allergy record are eligible for consideration of Penicillin Allergy De-labelling (PADL).
     2. A structured penicillin allergy history should be obtained from the patient, and other sources of information, by a doctor or pharmacist using the questions shown in Appendix 3.
     3. From the responses to these questions, use the validated allergy assessment tool shown in Appendix 4 to categorise the patient into one of the three allergy risk categories: ‘Low risk appropriate for direct de-label on history alone’, ‘Low risk eligible for direct oral challenge (DOC) test’ or ‘High risk’. NB the assessment tool needs to be viewed in colour. It is available via Microguide, accessed in the mobile phone app or via the documents library.
     4. Cut and paste the questions and the answers given into a ‘Pharmaceutical Care Plan’ note in EPMA with a title that reflects the outcome of the allergy risk assessment. Choose one of the following titles:
        1. “PADL HIGH” – high risk allergy history as per decision tool.
        2. “PADL LOW DDL” - low risk allergy history as per decision tool, eligible for direct de-label (removal of penicillin allergy record after allergy history reconciliation).
        3. “PADL LOW DOC” - low risk allergy history as per decision tool, eligible for Direct Oral Challenge.
        4. “PADL unable to obtain history (revisit)” - if the reason for non-obtaining history is likely to resolve during inpatient stay.
        5. “PADL unable to obtain history”- if the reason for non-obtaining history is likely permanent.
     5. High-risk patients are excluded at this stage with appropriate sign posting and referred for specialist allergy assessment via the outpatient allergy services in Plymouth, (see section 2.4), where appropriate.
     6. If the patient is assessed as low risk, then follow the process in the appropriate de-label pathway below (either DDL or DOC). If there is evidence that the patient has received a penicillin since the index reaction use Appendix 5 to determine appropriateness for DDL and follow DDL process below.
  2. **Direct de-label (DDL: de-label on history alone after allergy history reconciliation)**
     1. Counsel the patient on the risks and benefits of penicillin allergy records using the Information sheet in Appendix 6.
     2. With the patient’s consent, remove the incorrect penicillin allergy record from EPMA entirely or move it to the intolerance section of EPMA, if more appropriate to do so.
     3. It is good practice to add a note to EPMA stating that the patient’s allergy history has been removed, stating the history given by the patient and why it was removed. This will potentially help prevent re-labelling of the penicillin allergy during future episodes of care.
     4. Provide the patient with the leaflet in Appendix 7.
     5. If the GP records show a penicillin allergy record, then notify the GP of the amended allergy status either by emailing the letter in Appendix 8 with the patient’s details (preferred option) or adding a EPMA note ‘note to appear in GP letter’ stating the history given by the patients and the reason for de-label e.g. “Patient [name] reports an intolerance to penicillin (nausea) so we have removed this from their drug allergy box and prescribed amoxicillin for their CAP, which was well tolerated.”
  3. **Direct Oral Challenge (DOC)**
     1. Counsel the patient on the risks and benefits of penicillin allergy records and the risks and benefits of DOC using the Information sheet in Appendix 6.
     2. Consent the patient to an oral challenge test (single oral dose of index penicillin or amoxicillin if index penicillin unknown). Verbal patient consent needs will be sought prior to prescribing the dose of penicillin.
     3. The responsible clinician for the patient must provide consent for their patient to have an oral challenge dose.
     4. Check the patient does not meet any exclusion criteria for a direct oral challenge test (see list below).
     5. Exclusion criteria:
     - Pregnancy.
     - Cognitive impairment.
     - Where a collateral history cannot be obtained.
     - History of drug-associated anaphylaxis or oedema.
     - History of severe cutaneous adverse reactions.
     - History of acute kidney injury or severe liver impairment associated with antibiotic therapy.
     - Haemodynamic instability (defined as BP or HR outside normal range).
     - History of brittle / or severe asthma or has (had a course of steroids in the past 3 months for asthma).
     - Unstable coronary artery disease.
     - Uncontrolled heart failure.
     - Requiring oxygen to maintain oxygen saturations above 92% (relative contraindication, to be assessed in the context of the acuity of illness).
     - Antihistamines if cannot be withheld on day of testing.
     1. Discuss any uncertainties you have about challenge testing with either a medical microbiologist, infectious diseases consultant or antimicrobial stewardship pharmacist.
     2. Follow the DOC protocol in Appendix 9.
     3. Oral challenge testing must only be undertaken in an inpatient environment on the ward with access to anaphylaxis drugs and expertise in anaphylaxis management. Patients must be closely observed and must not leave the ward during testing.
     4. Ward nursing team or challenge test healthcare worker (pharmacist or doctor) to perform basic observations as per DOC protocol (Appendix 9) before, and after the DOC.
     5. If there is no evidence of a reaction one hour post direct oral challenge, then the allergy records should be amended, with the patient’s consent, and the GP notified of the removal of their penicillin allergy label.
     6. It is good practice to add a note to EPMA stating that the patient’s allergy history has been removed stating the history given by the patient and why it was removed. This will potentially help prevent re-labelling of the penicillin allergy during future episodes of care.
     7. If the GP records show a penicillin allergy record, then notify the GP of the amended allergy status either by emailing the letter in Appendix 8 with the patients details or adding a ‘note to appear in GP letter’ EPMA note stating the history given by the patients and the reason for de-label e.g. “[Patient name] reports a penicillin allergy but upon questioning patient, the patient reports a benign skin reaction with penicillin >10 years ago. After tolerating a test dose of amoxicillin, we have removed this allergy record. The patient tolerated a 5-day course of amoxicillin during current inpatient stay”.
     8. Inform the patient of their negative test result and counsel the patient of the 1-2% risk of a delayed benign skin reaction because of the penicillin exposure. Give the patient information leaflet in Appendix 7.
     9. In the unlikely event the patient has a positive reaction (examples of potential manifestations provided in Appendix 10) to the oral dose of penicillin then give the patient the information in Appendix 11.
     10. Notify the GP of the confirmed positive allergy status using the letter in Appendix 12 or add a ‘note to appear in discharge letter’ EPMA note stating the history given by the patients and the symptoms experienced that are in keeping with a positive allergic reaction e.g., “[Patient name] reports a penicillin allergy but upon questioning patient, the patient reports a benign skin reaction with penicillin >10 years ago. After exposure to amoxicillin [patient name] experienced a maculo-papular rash after 5 days. The patient is to retain their penicillin allergy status.”
  4. **Referral to allergy services (high risk)**
     1. Patients meeting criteria for specialist allergy assessment can be referred by their GP to outpatient allergy services in Plymouth, in accordance with NICE Drug Allergy Guideline (CG183) and local referral guidelines (see link below). These include patients with a history of:
     2. Suspected anaphylaxis.
     3. Non-immediate non-severe cutaneous reaction where that class of drug is considered essential to management.
     4. A severe non-immediate cutaneous reaction.
     5. Beta lactam allergy when (a) beta lactams are considered essential for management (b) there is likely to be frequent need for beta-lactam antibiotics in the future (e.g., recurrent bacterial infections or immune deficiency) (c) there is suspected allergy to at least one other class of antibiotics in addition to beta lactams.
     6. Local referral document here: <https://rms.cornwall.nhs.uk/rms/primary_care_clinical_referral_criteria/allergy>
  5. **Documentation of confirmed allergy status**
     1. If allergy confirmed to be high risk, then update allergy record on EPMA and ensure the allergy record documented on EPMA reflects the patient history given. If it does not, then update EPMA.
     2. Ensure the GP record reflects the allergy status with correct allergy history documented.
     3. If it does not, then add a note for GP (“note to appear in discharge summary” via EPMA).
  6. **Task specific responsibilities**
     1. The ward teams (either a medication optimisation pharmacy technician, ward pharmacist, antibiotic stewardship pharmacy technician, antibiotic pharmacist, or doctor) will take a structured penicillin allergy history using the set allergy history questions in this document and document the outcome in a Pharmaceutical Care Plan (PCP) note.
     2. Ward pharmacists, antibiotic pharmacists and doctors can remove penicillin allergy record via direct de-label if they feel competent to do so. (Please see suggested education resources in section 2.8).
     3. Ward pharmacists, antibiotic pharmacists and doctors can decide whether a patient is eligible for a direct oral challenge test using the risk stratification tool in this document, if they deem themselves competent to do so. (Please see suggested education resources in section 2.8).
     4. Prescribing a direct oral challenge dose can be done by a non-medical prescribing pharmacist or doctor with permission from the patient’s responsible clinician.
     5. The post-dose observations should ideally be carried out by a trained nurse but can be undertaken by others including healthcare assistants, medical and nursing students, ward pharmacists and ward doctors.
  7. **Testing environment**

Direct oral challenge testing must be done in a ward with access to trained medical and nursing staff and with access to the resuscitation team in the rare event the patient has a severe reaction.

- 1. **Education resources on penicillin allergy de-labelling for non-allergists**
     1. In the electronic staff record (ESR) select ‘learning certification’ and search ‘penicillin’ for education module on direct de-label on history alone.
     2. British Society of Antimicrobial Chemotherapy have a Massive Open Online Module on penicillin allergy (6 hours of learning over three weeks).
     3. Journal Article ‘Evaluation and Management of Penicillin Allergy A Review’ by Erica S. Shenoy, Theresa Rowe, Kimberly G. Blumenthal. JAMA. 2019;321(2):188-199.doi:10.1001/jama.2018.19283.

## Monitoring compliance and effectiveness

| Information Category | Detail of process and methodology for monitoring compliance |
| --- | --- |
| **Element to be monitored** | The number of patients risk assessed, the appropriateness of the allergy risk assessment, the numbers of patients meeting high risk and low risk criteria and the numbers safely de-labelled by both DDL and DOC. |
| **Lead** | Neil Powell and the antimicrobial stewardship team. |
| **Tool** | Monitored as part of an NIHR funded doctoral fellowship initially (first 6-12 months), then monitored as part of antimicrobial stewardship activity in the hospital. |
| **Frequency** | Ongoing audit, reported monthly. |
| **Reporting arrangements** | Antimicrobial Stewardship Management Committee (ASMC).  Medicines Practice Committee (MPC). |
| **Acting on recommendations and Lead(s)** | The ASMC will lead on subsequent recommendations made by the MPC. |
| **Change in practice and lessons to be shared** | Required changes to practice will be identified and actioned within a month. A lead member of the team will be identified to take each change forward where appropriate. Lessons will be shared with all the relevant stakeholders. |

## Equality and Diversity

- 1. This document complies with the Royal Cornwall Hospitals NHS Trust service Equality and Diversity statement which can be found in the [Equality Diversity And Inclusion Policy](https://doclibrary-rcht.cornwall.nhs.uk/DocumentsLibrary/RoyalCornwallHospitalsTrust/HumanResources/EqualityDiversityAndInclusionPolicy.pdf) or the [Equality and Diversity website](http://intranet-rcht.cornwall.nhs.uk/shelf/equality-and-diversity/).
  2. Equality Impact Assessment

The Initial Equality Impact Assessment Screening Form is at Appendix 2.

## Appendix 1. Governance Information

| Information Category | Detailed Information |
| --- | --- |
| **Document Title:** | Penicillin Allergy De-Labelling in Adult Inpatients Clinical Guideline V1.1 |
| **This document replaces (exact title of previous version):** | New Document |
| **Date Issued/Approved:** | 27 October 2023 |
| **Date Valid From:** | February 2023 |
| **Date Valid To:** | February 2023 |
| **Directorate / Department responsible (author/owner):** | Neil Powell, Consultant Antimicrobial Pharmacist. |
| **Contact details:** | 01872 252593  [rcht_penicillin@nhs.net](mailto:rcht_penicillin@nhs.net) |
| **Brief summary of contents:** | Penicillin allergy de-labelling guideline. |
| **Suggested Keywords:** | Penicillin, penicillin allergy, de-label, de-labelling. |
| **Target Audience:** | **RCHT:** Yes  **CFT:** No  **CIOS ICB:** No |
| **Executive Director responsible for Policy:** | Chief Medical Officer |
| **Approval route for consultation and ratification:** | Medicine Practice Committee (MPC) |
| **Manager confirming approval processes:** | Richard Andrzejuk |
| **Name of Governance Lead confirming consultation and ratification:** | Kevin Wright |
| **Links to key external standards:** | Start smart then focus: antimicrobial stewardship toolkit for inpatient care settings. Updated 12 September 2023. <https://www.gov.uk/government/publications/antimicrobial-stewardship-start-smart-then-focus/start-smart-then-focus-antimicrobial-stewardship-toolkit-for-inpatient-care-settings> |
| **Related Documents:** | BSACI guideline for the set-up of penicillin allergy de-labelling services by non-allergists working in a hospital setting https://doi.org/10.1111/cea.14217. |
| **Training Need Identified?** | Yes. Education and training will be delivered by the antimicrobial stewardship pharmacists to ward pharmacists, doctors, and nurses.  Signposting to education resources given in the guideline above see “2.8 Education resources on penicillin allergy de-labelling for non-allergists”. |
| **Publication Location (refer to Policy on Policies – Approvals and Ratification):** | Internet and Intranet |
| **Document Library Folder/Sub Folder:** | Clinical / Pharmacy |

**Version Control Table**

| **Date** | **Version Number** | **Summary of Changes** | **Changes Made by** |
| --- | --- | --- | --- |
| October 2023 | V1.0 | Initial issue | Neil Powell Consultant Antimicrobial Pharmacist |

**All or part of this document can be released under the Freedom of Information Act 2000.**

**All Policies, Strategies and Operating Procedures, including Business Plans, are to be kept for the lifetime of the organisation plus 6 years.**

**This document is only valid on the day of printing.**

**Controlled Document.**

This document has been created following the Royal Cornwall Hospitals NHS Trust [The Policy on Policies (Development and Management of Knowledge Procedural and Web Documents Policy)](https://doclibrary-rcht.cornwall.nhs.uk/DocumentsLibrary/RoyalCornwallHospitalsTrust/HealthInformatics/CorporateAndHealthRecords/ThePolicyOnPoliciesDevelopmentAndManagementOfKnowledgeProceduralAndWebDocumentsPolicy.pdf). It should not be altered in any way without the express permission of the author or their Line Manager.

**Appendix 2. Equality Impact Assessment**

**Section 1: Equality Impact Assessment (EIA) Form**

The EIA process allows the Trust to identify where a policy or service may have a negative impact on an individual or particular group of people.

For guidance please refer to the Equality Impact Assessment Policy (available from the document library) or contact the Equality, Diversity, and Inclusion Team [rcht.inclusion@nhs.net](mailto:rcht.inclusion@nhs.net)

| **Information Category** | **Detailed Information** |
| --- | --- |
| **Name of the strategy / policy / proposal / service function to be assessed:** | Penicillin Allergy De-Labelling in Adult Inpatients Clinical Guideline V1.1 |
| **Directorate and service area:** | Pharmacy, Clinical Support. |
| **Is this a new or existing Policy?** | New |
| **Name of individual completing EIA** (Should be completed by an individual with a good understanding of the Service/Policy): | Neil Powell Consultant Antimicrobial Pharmacist |
| **Contact details:** | 01872 252593 |

| **Information Category** | **Detailed Information** |
| --- | --- |
| 1. **Policy Aim - Who is the Policy aimed at?**   (The Policy is the Strategy, Policy, Proposal or Service Change to be assessed) | Doctors, nurses, medicines optimization pharmacy technicians and pharmacists. |
| 1. **Policy Objectives** | To remove incorrect penicillin allergy records from patient medical notes. |
| 1. **Policy Intended Outcomes** | More patients receiving first line antimicrobial agents. |
| 1. **How will you measure each outcome?** | Audit. |
| 1. **Who is intended to benefit from the policy?** | Patients, healthcare systems, wider society. |
| **6a. Who did you consult with?**  (Please select Yes or No for each category) | - Workforce: Yes - Patients/ visitors: Yes - Local groups/ system partners: No - External organisations: No - Other: No |
| **6b. Please list the individuals/groups who have been consulted about this policy.** | **Please record specific names of individuals/ groups:**  Medical consultants, intensive care consultants.  Medicine Practice Committee (MPC). |
| **6c. What was the outcome of the consultation?** | Positive, agreed. |
| **6d. Have you used any of the following to assist your assessment?** | **National or local statistics, audits, activity reports, process maps, complaints, staff, or patient surveys:**  Staff |

**7. The Impact**

Following consultation with key groups, has a negative impact been identified for any protected characteristic? Please note that a rationale is required for each one.

Where a negative impact is identified without rationale, the key groups will need to be consulted again.

| **Protected Characteristic** | **(Yes or No)** | **Rationale** |
| --- | --- | --- |
| **Age** | No |  |
| **Sex** (male or female) | No |  |
| **Gender reassignment** (Transgender, non-binary, gender fluid etc.) | No |  |
| **Race** | No |  |
| **Disability** (e.g. physical or cognitive impairment, mental health, long term conditions etc.) | No |  |
| **Religion or belief** | No |  |
| **Marriage and civil partnership** | No |  |
| **Pregnancy and maternity** | No |  |
| **Sexual orientation** (e.g. gay, straight, bisexual, lesbian etc.) | No |  |

**A robust rationale must be in place for all protected characteristics. If a negative impact has been identified, please complete section 2. If no negative impact has been identified and if this is not a major service change, you can end the assessment here.**

I am confident that section 2 of this EIA does not need completing as there are no highlighted risks of negative impact occurring because of this policy.

Name of person confirming result of initial impact assessment: Neil Powell Consultant Antimicrobial Pharmacist.

**If a negative impact has been identified above OR this is a major service change, you will need to complete section 2 of the EIA form available here:**[Section 2. Full Equality Analysis](http://doclibrary-rcht-intranet.cornwall.nhs.uk/DocumentsLibrary/RoyalCornwallHospitalsTrust/ChiefExecutive/Templates/Section2FullEqualityAnalysis.docx)

**Appendix 3. Penicillin allergy assessment questions**

The answers to the questions below will be used with the decision support tool below to decide whether the patient is de-labelled on history alone, is offered an oral challenge test or whether no intervention is able to be offered. Those meeting criteria for allergist referral will be considered for allergist referral if they meet the local referral criteria (below). Cut and paste the questions and the text below into an EPMA Pharmaceutical care plan note with ‘PADL’ as the title.

1. **Which penicillin did you react to?**
2. **Do you remember the details of the reaction?**
3. **How many hours after having your first dose of the antibiotic did the reaction occur?**
4. **How many years ago did the reaction occur?**
5. **How was the reaction managed? What was the outcome?**
6. **Which other antibiotics have you tolerated post reaction?**

Penicillin allergy history taken by: (name and job role)

Date

Risk assessment categorisation:

Proposed action:

Risk assessment person (name and job role)

Date

**Appendix 4. Validated Allergy Assessment Tool**

**Appendix 5. Managing patients who have tolerated a penicillin since the index reaction**

| **Scenario** | **Recommendation** | **Explanation** |
| --- | --- | --- |
| A history consistent with an IgE mediated reaction to a known penicillin and has tolerated the same penicillin or amoxicillin since the index reaction. | De-label on history alone. | Tolerating the index penicillin rules out IgE mediated reactions. Tolerating amoxicillin rules out IgE reaction to the beta-lactam ring a the most common R1 side chain allergen. Amoxicillin is the penicillin used to definitively rule out IgE allergic reactions during formal allergy testing. |
| A history consistent with an IgE mediated reaction to a known penicillin or unknown penicillin and has tolerated a penicillin antibiotic since but it was not the index penicillin or amoxicillin. | Cannot de-label based on the fact they have since tolerated a penicillin. | IgE mediated reactions can be due to the beta-lactam ring or the R1 side chain. Selective reactivity can occur to amoxicillin, piperacillin-tazobactam and flucloxacillin R1 side chains. American studies suggest selective IgE mediated allergy to amoxicillin is very rare whereas European studies suggest it may be between 25-50% of patients with positive skin tests have selective allergy to amoxicillin. |
| A history consistent with a low-risk allergy history that meets criteria for a DOC test but has tolerated any penicillin / unknown penicillin since the index reaction. | De-label on history alone. | Low risk allergy histories that meet criteria for DOC are not likely to re-react to penicillin when challenged. Ruling out beta-lactam ring sensitivity is sufficient in these patients. |
| An allergy history consistent with a severe delayed reaction (Gell and Coombs types 2-4) and includes DILI, cytopenia, AIN, AGEP, DRESS, SJS/TEN and tolerated any penicillin or unknown penicillin since index reaction. | Avoid all beta-lactams | High risk allergy group and little is understood about the best method to de-label these patients. Prior tolerance does not rule out severe delayed reactions on subsequent exposure. |

**Appendix 6.** [**Penicillin allergy de-labelling (RCHT 2060)**](https://doclibrary-rcht.cornwall.nhs.uk/DocumentsLibrary/RoyalCornwallHospitalsTrust/PatientInformation/Pharmacy/RCHT2060PenicillinAllergyDelabelling.pdf)


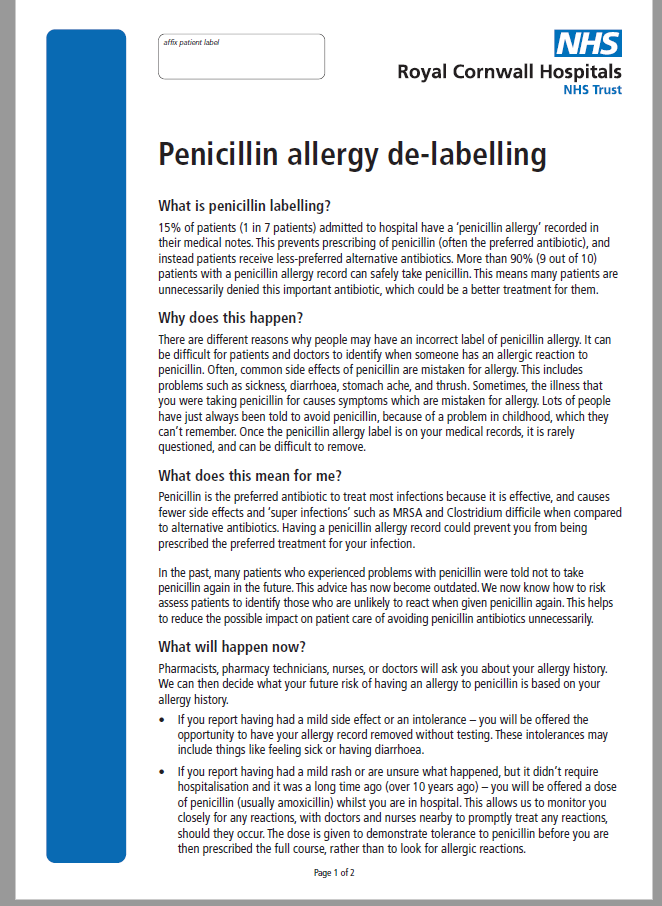


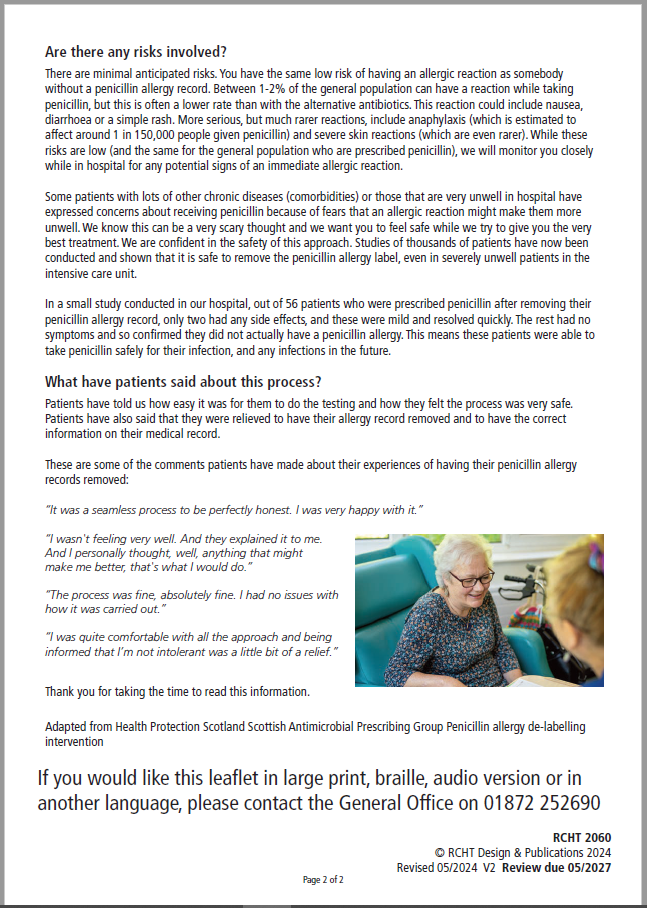


**Appendix 7.** [**You do NOT have an allergy to penicillin (RCHT 2061)**](https://doclibrary-rcht.cornwall.nhs.uk/DocumentsLibrary/RoyalCornwallHospitalsTrust/PatientInformation/Pharmacy/RCHT2061YouDoNotHaveAnAllergyToPenicillin.pdf)


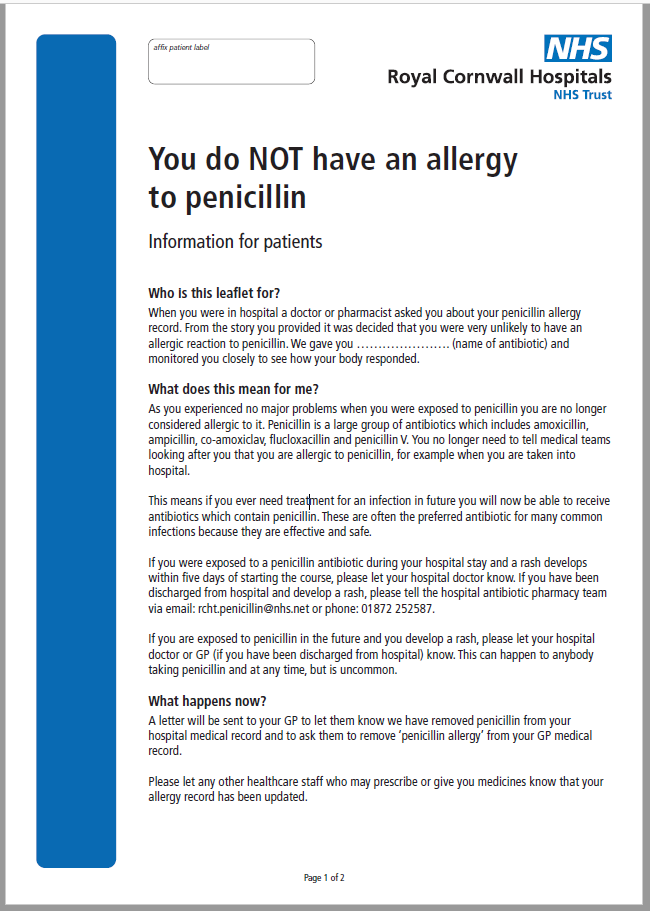


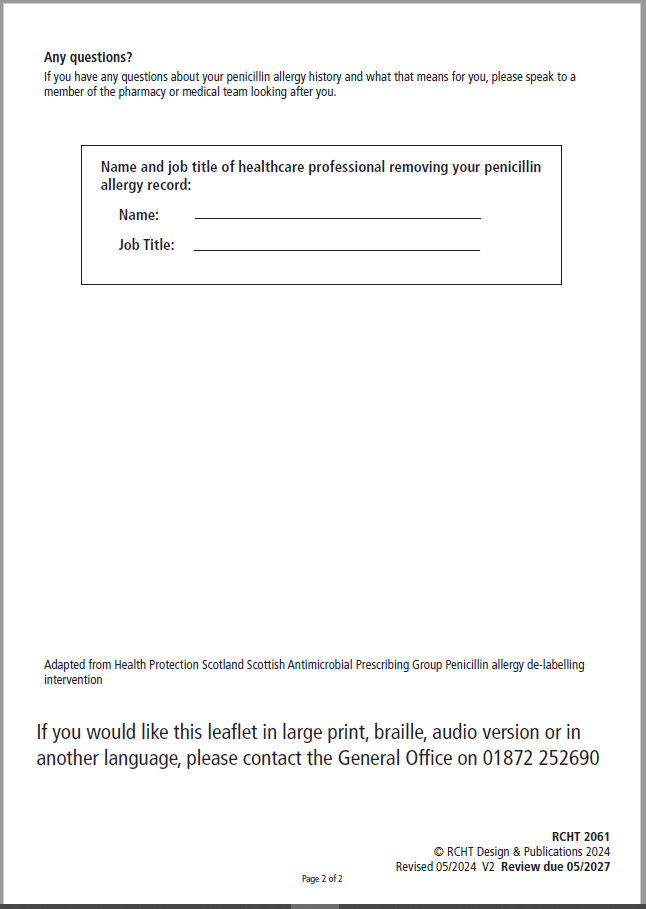


**Appendix 8. Penicillin Allergy Record – Remove Penicillin Allergy Label**

Date [today]

Patient name [xxxxxx]

Patient NHS no [xxxxxxx]

Dear Dr,

Your patient [John Smith] underwent assessment of their penicillin allergy label during a recent hospital attendance.

After review of their history, and discussion of the risk and benefits, we removed this allergy record from our hospital medical notes.

Patient gave a history as follows: [e.g. benign skin reaction in child hood / nausea].

Subsequent exposure to a penicillin antibiotic: [amoxicillin was given for 5 days / we did not expose the patient to penicillin on this admission].

Patient reports side effects: [the course was tolerated / we did not expose the patient to penicillin on this admission].

Based on this we can advise you that **there is no evidence to now support a “penicillin allergy” label.** We request that you remove this allergy label from your patient’s medical record and record details of the allergy test (antibiotic and date). This information should be shared with other healthcare providers within your Practice and their regular community pharmacy and general dental practitioner if possible.

The risk of allergic reaction to penicillin in a de-labelled patient is the same as that of the general population and **they can therefore receive penicillin antibiotics**. This is important for management of any future infections as penicillins are often recommended as first line therapy due to their effectiveness and lower risk of driving antimicrobial resistance.

**Your patient has also been informed that they can safely take penicillin-based antibiotics in future** and a copy of the information which they received after the test is included with this letter.

If you have any queries regarding the test process or outcome, please do not hesitate to contact me.

Thanks and best wishes.

Name and Grade

Enquiries contact:

Acknowledgments: letter adapted from Health Protection Scotland Scottish Antimicrobial Prescribing Group Penicillin allergy de-labelling intervention.

**Appendix 9. Direct oral challenge test procedure**

**Preparation**

1. Review the exclusion criteria for direct drug provocation testing.
2. Select the antibiotic to be used. In most cases this should be the penicillin antibiotic to which the patient had the adverse reaction. If the antibiotic is unknown, then amoxicillin is an appropriate choice.
3. Discuss the plan for an oral penicillin challenge with the patient and give them the patient information sheet.
4. Record in the case notes that consent has been obtained.

**Procedure for the direct drug provocation test**

1. Measure the patient’s observations (HR, BP, oxygen saturations, RR). If the patient has asthma, then measure peak expiratory flow rate (PEFR).
2. Prescribe and administer the antibiotic. Patient and observer to remain within the clinical area for 60 minutes.
3. Antibiotics should be administered as a single therapeutic oral dose: e.g. Amoxicillin 500mg.
4. Inform the patient to notify you immediately if they experience any adverse symptoms.
5. Ask the patient to report any symptoms and measure the patient’s observations (BP, pulse, Sp02, and PEFR if indicated) and at regular intervals e.g., at, 20 minutes, 40 minutes and 60 minutes and document.
6. Record any symptoms that the patient experiences.
7. If the patient reports any of the symptoms of a positive test (listed below) or they have a rising NEWS score, then the patient should be reviewed immediately by an appropriate senior member of staff.

**Post-procedure care**

1. Interpret the oral challenge with the following outcomes:
   1. Negative Test - no symptoms reported during the period of observation and patient’s NEWS score does not rise. Patient experiences isolated nausea or isolated itch without any of the other features of a positive test.
   2. Equivocal test - if there is doubt about the interpretation of the test then it should be discussed with a senior clinician and discussion with the local allergy service should be considered.
   3. Positive Test - patient experiences any of the following: itchy rash, breathing difficulties, facial swelling, hypotension, collapse, tongue swelling.
2. If the challenge is negative give the patient the patient information leaflet, record in the discharge letter and ask the patient’s GP to amend their allergy status on the practice records.
3. In case of late phase response, the patient must be instructed to call 111 or visit their local Emergency Department should they develop symptoms of dyspnoea, wheezing, dizziness, or severe pruritus.
4. If the challenge outcome is positive written and electronic record must clearly state this. The patient should be provided with the information leaflet and the GP informed of this outcome.

**Appendix 10. Signs and symptoms of potential allergy**

Signs and Symptoms of allergic reactions in various target organs. (Used with permission from the ALABAMA study Team).

**Signs and symptoms of potential allergy:**

Skin: Urticaria/Angioedema.

Flushing.

Erythematous pruritic rash.

Atopic dermatitis.

Gastro-intestinal tract: Pruritis and /or swelling of the lips, tongue, or oral mucosa.

Nausea.

Abdominal cramping or colic.

Vomiting or reflux.

Diarrhoea.

Respiratory tract: Nasal congestion.

Rhinorrhoea.

Pruritis/sneezing.

Laryngeal oedema, staccato cough and/or dysphonia.

Wheezing/ repetitive cough.

Dyspnoea.

Cardiovascular: Hypotension/shock.

Dizziness.

**Appendix 11.** [**Positive penicillin allergy testing result (RCHT 2062)**](https://doclibrary-rcht.cornwall.nhs.uk/DocumentsLibrary/RoyalCornwallHospitalsTrust/PatientInformation/Pharmacy/RCHT2062PositivePenicillinAllergyTestingResult.pdf)


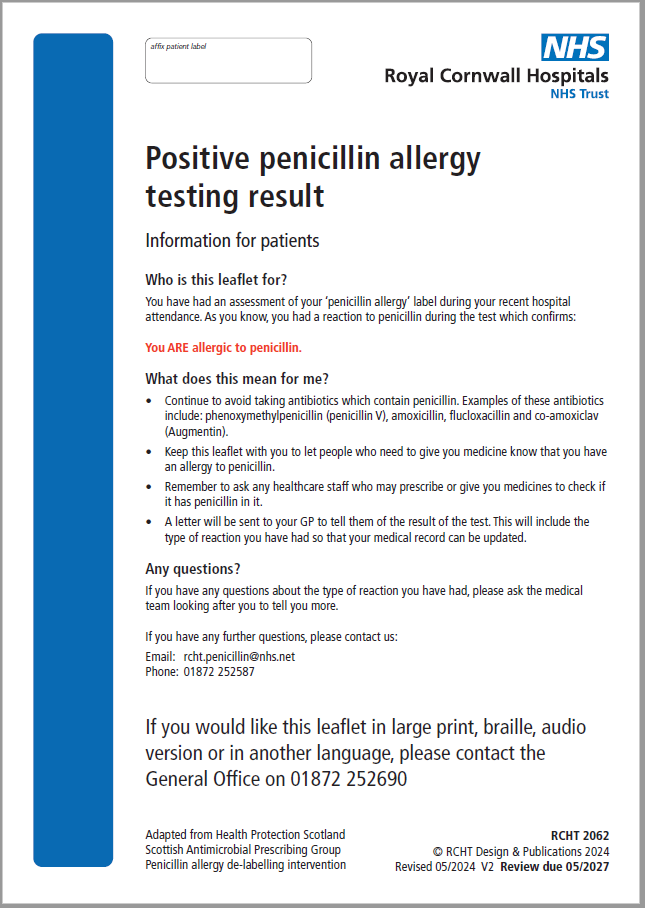


**Appendix 12. Penicillin Allergy Test – Reaction Confirmed**

Dear Dr XXXXXXXXXXXXXX,

Your patient XXXXXXXXX XXXXXXXXXXX underwent assessment of their penicillin allergy label during a recent hospital attendance.

After review of their history, and discussion of the risk and benefits, a supervised oral challenge was performed. A dose of 500mg of **amoxicillin / flucloxacillin** was administered on **[date]**.

**There was evidence of an allergic reaction,** as detailed below:

xx

Please record this description and date of observation in their medical record as confirmation of their allergy status. Please also ensure their allergy status is clearly documented in all future correspondence/records.

The patient has been informed that they **should not take penicillin-based antibiotics** in future. Examples of these antibiotics include phenoxymethylpenicillin (penicillin V), amoxicillin, flucloxacillin and co-amoxiclav (Augmentin). A copy of the information which your patient has received after the test is included with this letter.

Thanks and best wishes

Name/Grade

Enquiries/Contact:

Acknowledgments: letter adapted from Health Protection Scotland Scottish Antimicrobial Prescribing Group Penicillin allergy de-labelling intervention
